# Expression of the Blood-Group-Related Gene *B4galnt2* Alters Susceptibility to *Salmonella* Infection

**DOI:** 10.1371/journal.ppat.1005008

**Published:** 2015-07-02

**Authors:** Philipp Rausch, Natalie Steck, Abdulhadi Suwandi, Janice A. Seidel, Sven Künzel, Kirandeep Bhullar, Marijana Basic, Andre Bleich, Jill M. Johnsen, Bruce A. Vallance, John F. Baines, Guntram A. Grassl

**Affiliations:** 1 Institute for Experimental Medicine, Christian-Albrechts-University of Kiel, Kiel, Germany; 2 Max Planck Institute for Evolutionary Biology, Plön, Germany; 3 Models of Inflammation, Research Center Borstel, Borstel, Germany; 4 Department of Pediatrics, Division of Gastroenterology, Child and Family Research Institute, University of British Columbia, Vancouver, British Columbia, Canada; 5 Institute for Laboratory Animal Science, Hannover Medical School, Hannover, Germany; 6 Research Institute, Puget Sound Blood Center, Seattle, Washington, United States of America; 7 Department of Medicine, University of Washington, Seattle, Washington, United States of America; University of California, Davis, UNITED STATES

## Abstract

Glycans play important roles in host-microbe interactions. Tissue-specific expression patterns of the blood group glycosyltransferase β-1,4-N-acetylgalactosaminyltransferase 2 (*B4galnt2*) are variable in wild mouse populations, and loss of *B4galnt2* expression is associated with altered intestinal microbiota. We hypothesized that variation in *B4galnt2* expression alters susceptibility to intestinal pathogens. To test this, we challenged mice genetically engineered to express different *B4galnt2* tissue-specific patterns with a *Salmonella* Typhimurium infection model. We found *B4galnt2* intestinal expression was strongly associated with bacterial community composition and increased *Salmonella* susceptibility as evidenced by increased intestinal inflammatory cytokines and infiltrating immune cells. Fecal transfer experiments demonstrated a crucial role of the *B4galnt2*-dependent microbiota in conferring susceptibility to intestinal inflammation, while epithelial *B4galnt2* expression facilitated epithelial invasion of *S*. Typhimurium. These data support a critical role for *B4galnt2* in gastrointestinal infections. We speculate that *B4galnt2*-specific differences in host susceptibility to intestinal pathogens underlie the strong signatures of balancing selection observed at the *B4galnt2* locus in wild mouse populations.

## Author Summary

Human blood groups are among the oldest known genetic polymorphisms. It has been proposed that blood group variation is a byproduct of pathogen-driven selection, including in the gastrointestinal tract where blood-group-related genes are often variably expressed. The *B4galnt2* gene is responsible for the synthesis of the Sd(a)/Cad carbohydrate blood group antigen and displays variable tissue-specific expression patterns in wild mouse populations. Using an established model for *Salmonella* Typhimurium induced colitis, we found that loss of *B4galnt2* expression in the intestinal epithelium decreases susceptibility to infection. These effects were strongly associated with the influence of *B4galnt2* expression on the intestinal microbiota, whereby microbial diversity *prior* to infection was highly predictive of reduced inflammation and resistance to *Salmonella* Typhimurium infection. Additionally, *B4galnt2* expression in blood vessels also distinctly influenced intestinal phenotypes and *Salmonella* susceptibility. These data lend new insights into bacterial community diversity as an “extended phenotype” that can be mediated by host genetic variation at blood-group-related genes. This work further provides strong experimental evidence in support of a scenario of complex selection on the *B4galnt2* tissue-specific expression variants via host-microbe relationships and susceptibility to infectious disease.

## Introduction

The luminal surface of the intestinal mucosa is covered by distinct layers of highly glycosylated mucus that form a physical barrier between the intestinal microbial community and the host’s tissues. In addition to their important roles in host metabolism and signaling, glycans are known to contribute to the composition and physiology of the intestinal microbiota, thereby playing an important role in regulating microbe-host interactions [1]. Host glycans can contribute to a beneficial microenvironment for symbiotic microbes by providing carbohydrate sources or by serving as attachment sites [1–3], but glycans can in the same way also mediate pathogenic interactions [4, 5]. The patterns of intestinal carbohydrate structures, which vary along sites of the gastrointestinal tract, are the product of a combination of host glycosyltransferase expression programs as well as microbial influences [6, 7].

The genes responsible for synthesizing carbohydrate blood group antigens frequently display signatures of balancing selection and are implicated in the co-evolution of hosts and their pathogens [8]. A well-described example is the *FUT2* gene, which encodes an α-1,2-fucosyltransferase that directs the expression of the H antigen in mucosal tissues and bodily secretions. Homozygosity for loss-of-function *FUT2* mutations leads to loss of expression of ABO and H blood group glycans in secretions and is known as the “nonsecretor” phenotype, which is common in human populations [9]. Nonsecretor status has been implicated as a detrimental genetic risk factor for inflammatory disorders such as Crohn’s disease [10] and primary sclerosing cholangitis [11], while being positively associated with resistance to intestinal pathogens [12–14]. Glycosylation of the epithelium has recently been recognized as a direct immune cell mediated response to infection as a means to restore the protective functions of the microbial community and to ensure tissue homeostasis [15–17]. Glycans also mediate species specificity of pathogens. For example, the different associations of *Helicobacter* species *to* Lewis antigens in the canine gastric mucosa [18].

Gastrointestinal (GI) expression of the blood group glycosyltransferase β-1,4-N-acetylgalactosaminyltransferase 2 (*B4galnt2)*, which directs biosynthesis of a carbohydrate antigen similar to blood group A termed the Sd(a) [19] is conserved across vertebrates [20]. However, in mice there is a common allele which confers a tissue specific switch in *B4galnt2* expression from gut to blood vessels [21]. This allele is termed “*Modifier of von Willebrand Factor-1*” (*Mvwf1*) [22] because *B4galnt2* vascular expression leads to aberrant glycosylation of the vascular-derived blood coagulation factor von Willebrand factor (VWF), resulting in accelerated VWF clearance from circulation [23]. *Mvwf1* was first described in the RIIIS/J inbred mouse strain [22], and subsequent studies revealed RIIIS/J-like *B4galnt2* alleles, which confer the *B4galnt2* tissue-specific switch from gut (epithelial) to blood vessel (endothelial) expression, to be common in wild mouse populations [24]. Further, this variation appears to have been maintained in the mouse lineage for several million years despite the presumed detrimental effect of prolonged bleeding time, possibly due to a protective role in host-pathogen interactions [25]. A role for *B4galnt2*-glycans in intestinal host-microbe interactions is supported by the observation of significant alterations in the intestinal microbiota in *B4galnt2*-deficient mice [26]. Taken together, the prevalence of alleles conferring the tissue-specific switch in *B4galnt2* expression in mice, the strong signatures of selection observed at the *B4galnt2* locus in wild mouse populations and the altered resident microbiota found in *B4galnt2*-deficient mice support the hypothesis that variant tissue-specific *B4galnt2* expression alters susceptibility to enteric infections in mice.

To investigate the role of variant host *B4galnt2* expression in the context of intestinal infection, we challenged mice engineered to express *B4galnt2* in various tissue-specific patterns with a mouse model of the intestinal pathogen *Salmonella enterica* serovar Typhimurium (*S*. Typhimurium). Prior to- and during the course of infection, we examined histological and molecular markers of inflammation along with bacterial community profiles. We found that the composition of the intestinal microbiota was consistently influenced by the expression of *B4galnt2*-glycans, and that *B4galnt2*-associated intestinal microbial community profiles were predictive of- and responsible for susceptibility to *S*. Typhimurium infection. We demonstrate that mice deficient in intestinal *B4galnt2* expression developed significantly less pathology after *S*. Typhimurium infection, in concert with attenuated induction of pro-inflammatory cytokines and infiltration of immune cells. Furthermore, we find that vascular *B4galnt2* expression leads to decreased *Salmonella* colonization and increased inflammatory cytokine expression. Overall, our study elucidates a new role for this key host carbohydrate blood group antigen in the interplay between the host, commensals, and susceptibility to pathogen infections.

## Results

### *B4galnt2* expression influences susceptibility to *S*. Typhimurium-induced colitis

**Fig 1:**
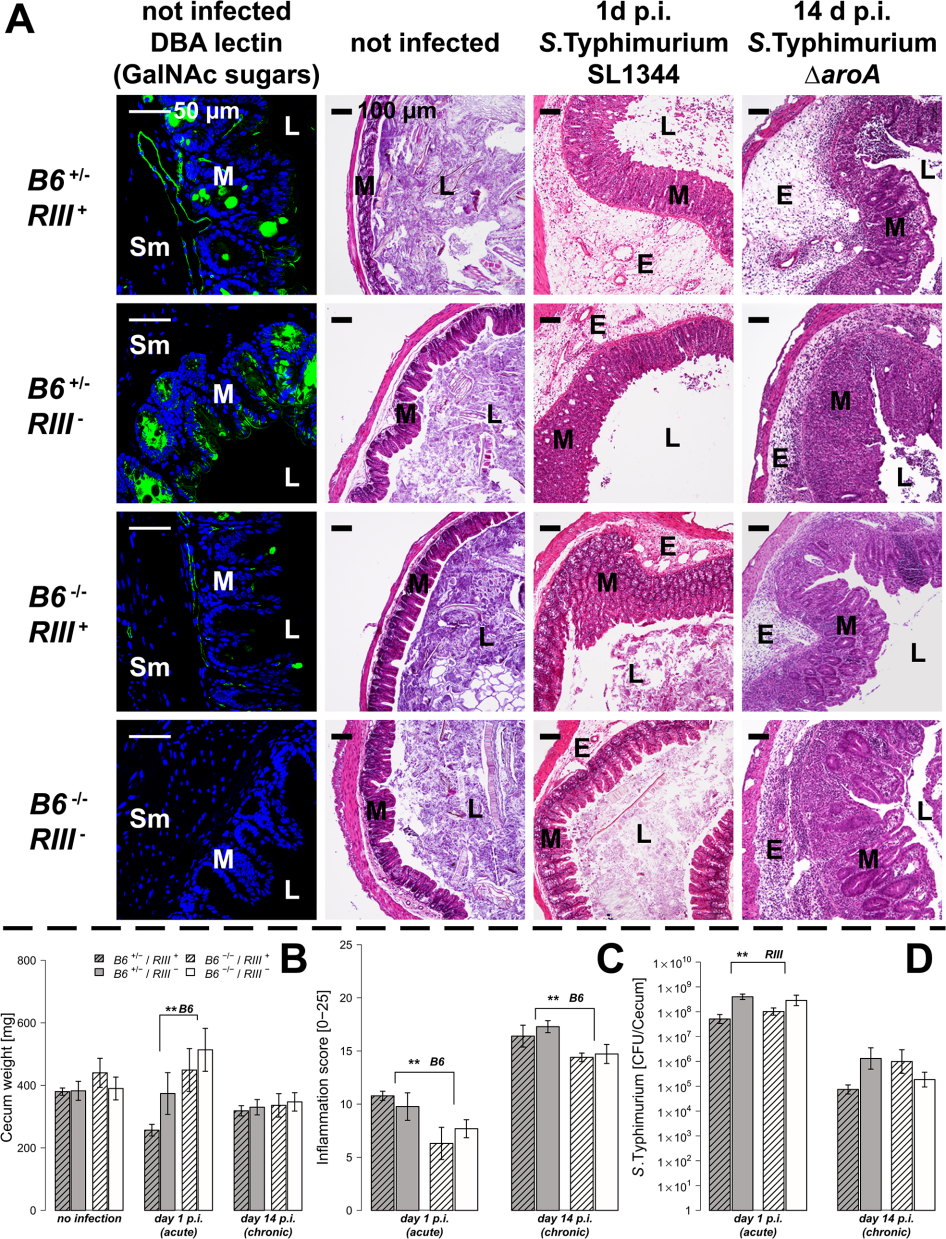
Tissue-specific expression of *B4galnt2* glycans influence susceptibility to *S*. Typhimurium-induced colitis. Mice were treated with streptomycin 24 h prior to infection with *S*. Typhimurium strain SL1344 for 24 h (acute) or with the attenuated strain *S*. Typhimurium Δ*aroA* for 14 days (chronic). (A) *B4galnt2* expression phenotype is characterized by GalNAc residues, stained for by Dolichus biflorus agglutinin (DBA). H&E staining of cecal sections illustrated higher numbers of cells in the lumen (L), an increased influx of inflammatory cells to mucosa (M) and submucosa (Sm), epithelial cell desquamation and the formation of submucosal edema (E) upon infection with *S*. Typhimurium (bar = 100 μm). (B) Cecal weight indicated a significant influence of intestinal *B4galnt2* glycans on S. Typhimurium induced colitis in the acute model (*B6*: *F*_1,49_ = 8.709, *P* = 0.0048; Linear model). (C) Histological scoring revealed higher inflammation in *B6* ^+/-^ compared to *B6* ^-/-^ mice (*B6*: *F*_1,49_ = 13.242, *P* = 0.0007; Linear model of X^4^ transformed inflammation scores). (D) Intestinal *S*. Typhimurium colonization was determined in tissue homogenates (*RIII*: *F*_1,49_ = 10.537, *P* = 0.0021; Linear model of log(CFU)). Data are presented as mean ± SEM, N = 9–19 per group in the acute model, N = 5–7 in the chronic model (# *P*<0.100, * *P*<0.050, ** *P*<0.010, *** *P*<0.001).

To test the hypothesis that expression of intestinal *B4galnt2* glycans influences host susceptibility to enteric pathogens, we used an established model for *S*. Typhimurium induced colitis [27]. Mice were bred to carry the desired combinations of alleles which express *B4galnt2* in the intestinal epithelium (*“B6*”: referring to the endogenous C57BL6/J allele), vascular endothelium (“*RIII”*: referring to the RIIIS/J-derived *Mvwf1* bacterial artificial chromosome transgene [21]), or lack a functional *B4galnt2* gene due to a targeted knock-out allele (“*B6* ^-/-^”: referring to the *B4galnt2* knock-out [23]). Twenty-four hours after streptomycin pre-treatment, mice were orally infected with *S*. Typhimurium SL1344 (“acute” infection, examined after 24 hours [28]) or the attenuated Δ*aroA* mutant (“chronic” infection, examined after 14 days [29]). None of the animals showed signs of inflammation or other pathology prior to infection. After infection in both the acute and chronic *Salmonella* models, mice expressing *B4galnt2* in the intestinal epithelium (*B6* ^+/-^ / *RIII* ^-^ and *B6* ^+/-^ / *RIII* ^+^) exhibited higher numbers of detached epithelial cells and neutrophils within the cecal lumen, increased inflammatory cell infiltration [29, 30] within the intestinal mucosa, and worsened submucosal edema in the ceca (Fig 1A). The dramatic reduction of cecum weight in infected *B6* ^+/-^ mice compared to *B6* ^*-/-*^ mice in acute *Salmonella* infection one day post infection (p.i.) indicated more severe disease [27] (Fig 1B). Accordingly, mice that did not express *B4galnt2* in the intestinal epithelium (*B6* ^-/-^) developed significantly less cecal inflammation in both the acute and chronic infection model (Fig 1C).

In order to evaluate *Salmonella* colonization, colony forming units (CFUs) were quantified from homogenized ceca. While *Salmonella* burdens were comparable between different *B4galnt2* intestinal epithelial-expressing genotypes (*B6*), *RIII* ^+^ (*B4galnt2-*endothelial expressing) animals exhibited lower *Salmonella* colonization in the acute *Salmonella* infections (Fig 1D). These results demonstrate a significant influence of intestinal epithelial *B4galnt2* expression on susceptibility to *Salmonella*-induced colitis, and an independent effect of vessel-specific *B4galnt2* expression on *Salmonella* burden. In contrast, infection of mice without prior streptomycin treatment resulted in equal bacterial organ colonization, organ weights, and elicited no intestinal inflammation regardless of the genotype of mice (S1 Fig). Due to the marked differences between mouse *B4galnt2* genotypes in the acute infection model, we performed further studies only in this model.

**Fig 2:**
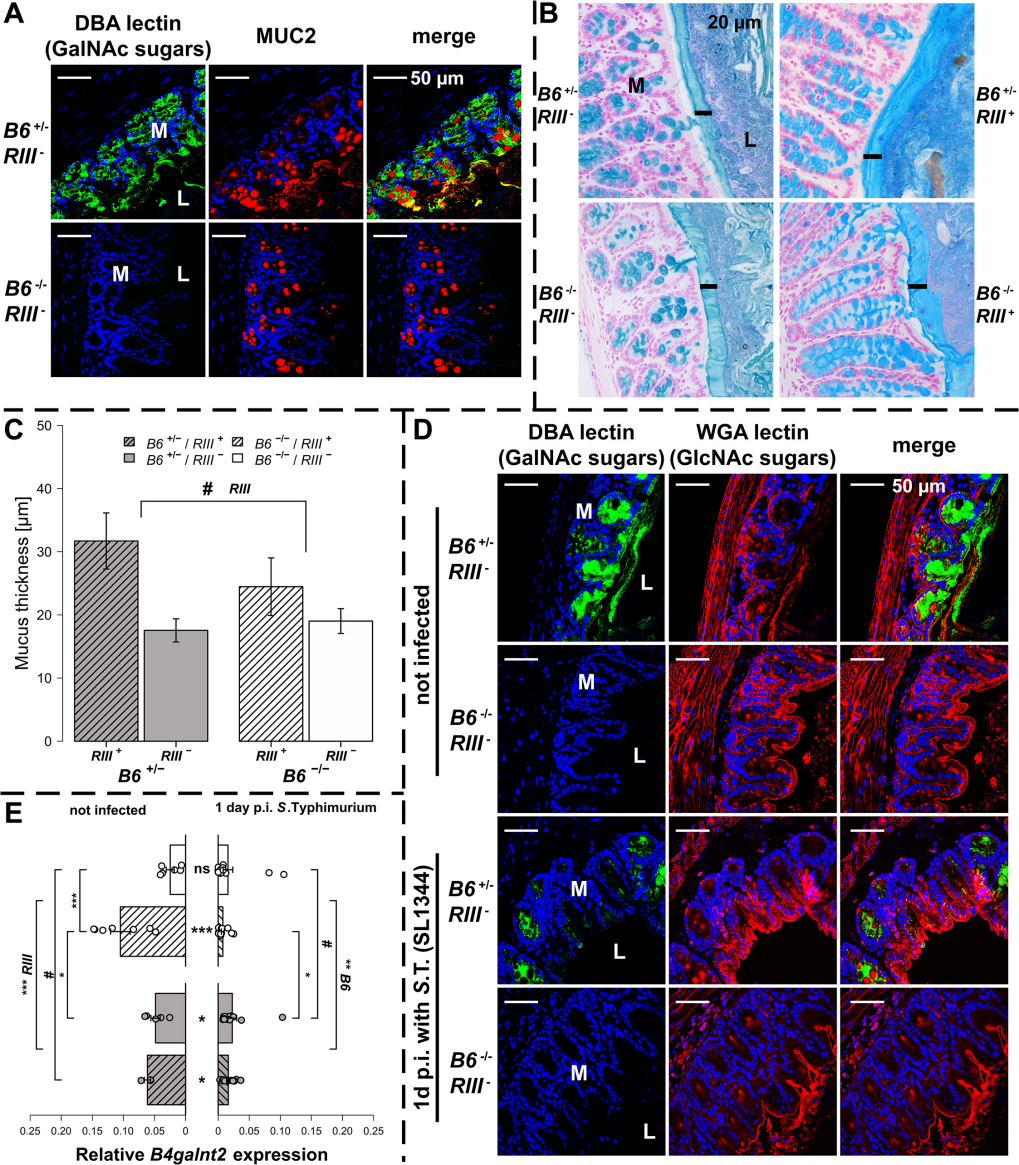
*B4galnt2* glycosylation in *S*. Typhimurium-induced colitis. (**A**) MUC2 (red) and DBA lectin (green) staining in formalin fixed cecal tissue sections (**B**) Acidic mucus was stained with alcian blue in Carnoy’s-fixed tissue sections (bar = 20 μm). (**C**) Mucus thickness was determined at five different regions within one animal from which mean values were analysed (N = 3–5; *Z* = -1.807, *P* = 0.0816; Wilcoxon test via Monte-Carlo resampling). (**D**) *B4galnt2* glycan residues (GalNAc) were stained with fluorescein labeled DBA (green) in formalin fixed cecal tissue sections before and 1 day p.i. with *S*. Typhimurium. GlcNAc residues were stained with Alexa633 labeled Wheat Germ Agglutinin (WGA) (red). (**E**) Relative expression of *B4galnt2* before and after infection with *S*. Typhimurium showing significant differences between *B6* and *RIII* genotypes before infection (*B6*: *F*_1,17_ = 0.216, *P* = 0.64779; *RIII*: *F*_1,17_ = 23.959, *P* = 0.00014; *B6*/*RIII*: *F*_1,17_ = 7.687, *P* = 0.01304 [pairwise comparisons- *B6* ^-/-^/*RIII* ^+^|*B6* ^-/-^/*RIII* ^-^: *P* = 0.00018, *B6* ^+/-^/*RIII* ^+^|*B6* ^-/-^/*RIII* ^-^: *P* = 0.08626, *B6* ^-/-^/*RIII* ^+^|*B6* ^+/-^/*RIII* ^-^: *P* = 0.02673]; Linear model and Tukey *post-hoc* test) and *B6* genotype differences after infection (*F*_1,53_ = 11.787, *P* = 0.001165). Infection has additional influence on *B4galnt2* expression (*Z* = 5.268, *P*<0.00001, Wilcoxon test via Monte-Carlo resampling), which is also genotype specific (*B6* ^+/-^/*RIII* ^+^: *Z* = 2.6458, *P*_*Bonferroni*_ = 0.01192, *B6* ^+/-^/*RIII* ^-^: *Z* = 2.6122, *P*_*Bonferroni*_ = 0.02832; *B6* ^-/-^/*RIII* ^+^: *Z* = 3.5496, *P*_*Bonferroni*_ = 0.00016, *B6* ^-/-^/*RIII* ^-^: *Z* = 2.0642, *P*_*Bonferroni*_ = 0.16132; Wilcoxon test via Monte-Carlo resampling; # *P* < 0.100, * *P* < 0.050, ** *P* < 0.010, *** *P* < 0.001; error bars indicate SEM).

*B4galnt2*-GalNAc residues have been shown to be detectable on the apical surface of intestinal epithelial cells [23, 26]. Immunohistochemical co-staining with *Dolichos biflorus* agglutinin (DBA) specifically detecting *B4galnt2-*derived β-1,4 linked GalNAc residues [21, 23] and MUCIN 2 (MUC2), the major secreted mucus protein in the large intestine, demonstrated a partial co-localization in goblet cells (Figs 2A and S2A). While MUC2 is considered to be glycosylated by B4GALNT2 [31], GalNAc residues were also detected in the intestinal mucosa of *Muc2*-deficient mice (S2B Fig), indicating the presence of other B4GALNT2-glycosylated substrates such as glycolipids [32, 33] and other glycoproteins [34–36]. To determine if *B4galnt2-*mediated glycosylation altered overall mucus thickness, which could make it easier for bacteria to cross the mucus layer and reach the epithelium, intestinal tissue of uninfected mice were fixed with Carnoy’s fixative, stained with alcian blue and the thickness of the dense inner mucus layer was determined. Although mucus thickness was not significantly affected by the lack of intestinal *B4galnt2* expression (*B6* ^-/-^), it did show slight differences between *RIII* ^+^ and *RIII* ^-^ (Fig 2B and 2C). Furthermore, less DBA lectin staining was observed in the cecal mucosa of *S*. Typhimurium infected mice on day one p.i. compared to uninfected mice (Fig 2D). In contrast to the DBA staining (GalNAc), the detection of N-Acetylglucosamine (GlcNAc) residues recognized by Wheat Germ Agglutinin (WGA) showed no clear difference after infection, suggesting the alteration of mucosal DBA lectin-reactive carbohydrate profiles that occur in response to *S*. Typhimurium infection did not affect substrates glycosylated by WGA-reactive GlcNAc (Fig 2D). *B4galnt2* gene expression was also down regulated upon infection (Fig 2E) which further corroborates the lectin staining results.

**Fig 3:**
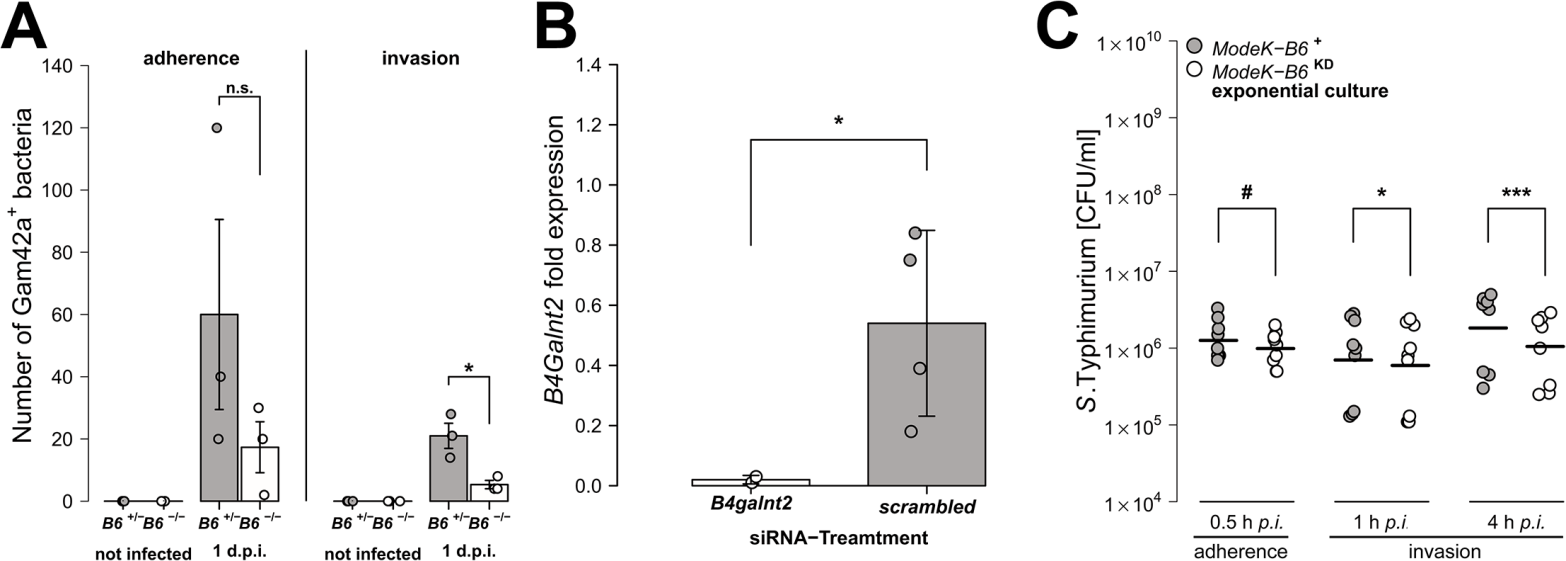
Epithelial *B4galnt2*-expression increases invasion by *S*. Typhimurium. (A) Carnoy’s fixed cecal sections were stained by FISH (Gam42a probe) to visualize bacteria. Measurement of *Salmonella* adherence (*t*_2.286_ = -1.349, *P* = 0.2954, unpaired t-test) and mucosa invasion (*t*_2.430_ = -3.681, *P* = 0.0491; bacterial counts in 10 high power fields per individual, N = 3; unpaired t-test). (B) *B4galnt2* expression in Mode-K cells after transfection with *B4galnt2* specific siRNA and scrambled siRNA relative to untreated cells (*t*_3.025_ = -3.3601, *P* = 0.0432; unpaired t-test). (C) *Salmonella* invasion of Mode-K cell cultures transfected with *B4galnt2* specific and scrambled siRNA, infected with *S*. Typhimurium. There is no significant effect of *B4galnt2* expression for adhesion of the bacteria to Mode-K cells (0.5 h: *F*_1,14_ = 3.133, *P* = 0.0985; LMM with experiment as random factor), while invasion of *S*. Typhimurium into Mode-K cells expressing *B4galnt2* was slightly better than into cells with *B4galnt2* knockdown (1 h: *F*_1,14_ = 7.644, *P* = 0.0152, 4 h: *F*_1,14_ = 26.336, *P* = 0.0002; LMM with experiment as random factor; # *P* < 0.100, * *P* < 0.050, ** *P* < 0.010, *** *P* < 0.001; error bars indicate SEM).

To test the direct effect of *B4galnt2* expression on *Salmonella’s* interaction with the cecal epithelium, we performed both FISH staining of cecal sections 1 day p.i. as well as *in vitro* experiments with the intestinal epithelial Mode-K cell line and siRNA-mediated knockdown of *B4galnt2* expression. Bacteria were stained by FISH using the Gam42a probe, which stains γ-Proteobacteria. In our experience virtually all Gam42a positive bacteria reaching the tissue in the streptomycin model at day 1 p.i. are *Salmonella*. Bacteria were counted if they were adherent to epithelial cells or invaded into the tissue in ten high power fields per cecal section. While adherent *Salmonella* were not significantly different in *B6* ^+/-^ mice compared to *B6* ^-/-^ mice, significantly more *Salmonella* were found to have invaded into the tissue of *B6* ^+/-^ mice (Fig 3A). To further investigate whether *B4galnt2* expression influences the interaction of *Salmonella* with epithelial cells, we used the intestinal epithelial Mode-K cell line and siRNA-mediated knockdown of *B4galnt2* (knockdown efficiency: 96%; Fig 3B). Adhesion and invasion assays showed that knockdown of *B4galnt2* expression does not significantly influence adhesion of *Salmonella* to epithelial cells (Fig 3C). However, invasion of *S*. Typhimurium into *B4galnt2*-expressing cells is slightly, but significantly increased relative to *B4galnt2*-knockdown cells (Fig 3C). This data shows that epithelial expression of *B4galnt2*- both *in vitro* and *in vivo*- directly facilitates invasion by *Salmonella*.

### Intestinal epithelial *B4galnt2* glycans are associated with elevated cytokine levels and higher numbers of inflammatory/immune cells after *S*. Typhimurium-induced colitis

**Fig 4:**
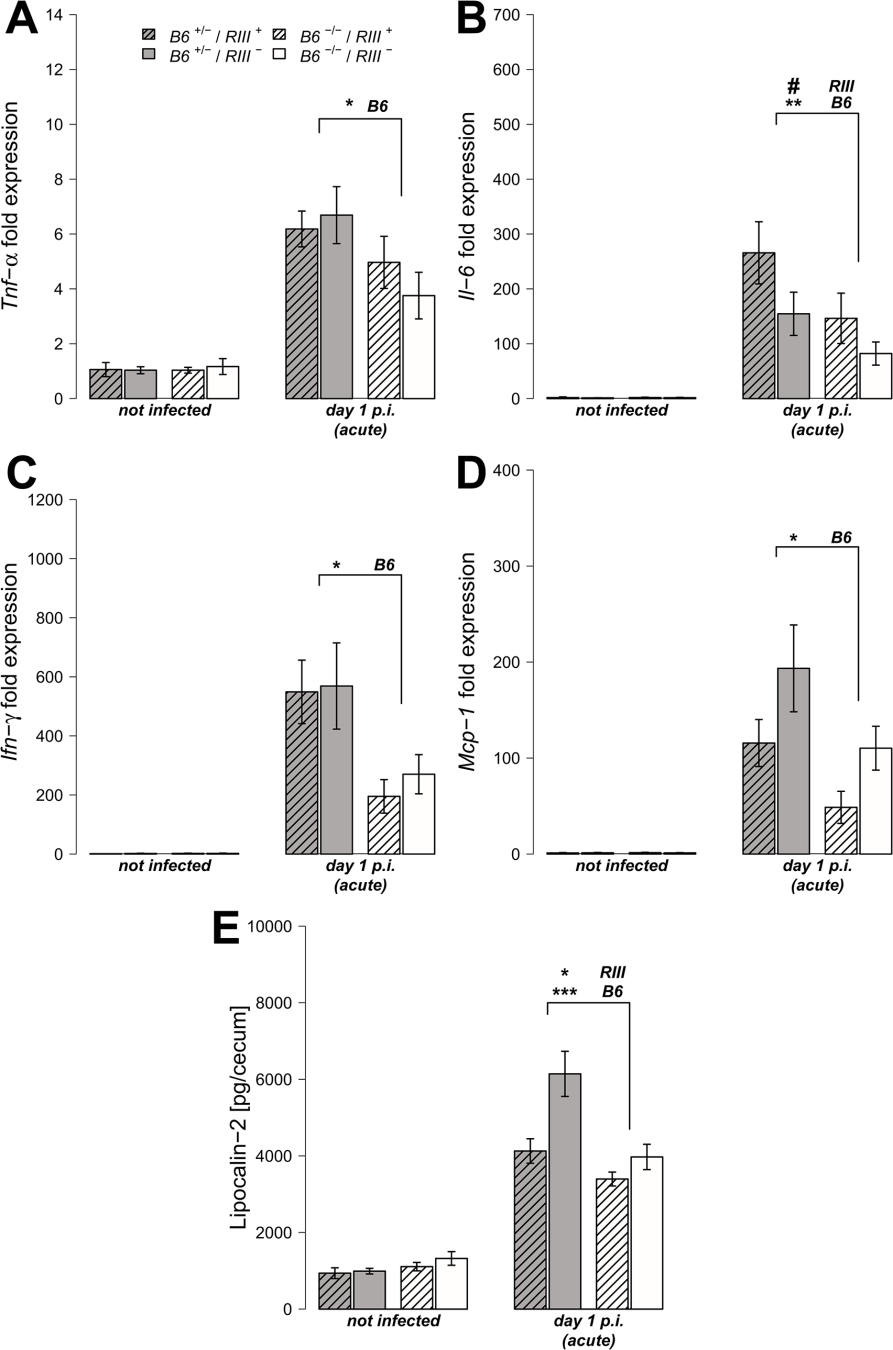
*B4galnt2*-dependent immune response after *S*. Typhimurium infection. (A-D) Relative gene expression of *Tnf-α*, *Il-6*, *Inf-γ* and *Mcp-1* was determined by RT-qPCR analysis. Values were normalized to *Gapdh* and *Hprt* and calculated as fold expression compared to the non-infected samples of each respective genotype. (E) Lipocalin-2 levels were measured by ELISA in supernatants of cecal homogenates (N = 3–11 per group) before- and one day p.i. with *S*. Typhimurium, showing a clear increase with infection (*Z* = -2.219, *P* = 0.0261; Wilcoxon test via Monte-Carlo resampling) and differences between *B6* and *RIII* genotypes (S1 Table, # *P* < 0.100, * *P* < 0.050, ** *P* < 0.010, *** *P* < 0.001; error bars indicate SEM).

We analyzed the transcript levels of pro-inflammatory cytokine genes in cecal tissues both prior to and after *S*. Typhimurium infection, focusing on those cytokines known to be induced early in *Salmonella*-triggered inflammation and associated with control of infection [37, 38]. The transcripts for the cytokines *Tumor necrosis factor-α* (*Tnf-α*), *Interleukin-6* (*Il-6*), *Interferon-γ* (*Ifn-γ*) and *Monocyte chemotactic protein-1* (*Mcp-1*) were elevated in all mice after infection, but to a significantly higher degree in *B6* ^+/-^ mice compared to *B6* ^-/-^ mice one day p.i. (Fig 4A–4D; *Tnf-α*: *Z* = -2.123, *P* = 0.0336; *Il-6*: *Z* = -2.458, *P* = 0.0138; *Ifn-γ*: *Z* = -2.417, *P* = 0.0147; *Mcp-1*: *Z* = -2.219, *P* = 0.0261; Wilcoxon test via Monte-Carlo resampling). Protein levels of Lipocalin-2 (LCN-2), a molecule implicated in antimicrobial defense and innate immunity [39, 40], were also increased in cecal tissue homogenates in *B6* ^+/-^ mice compared to *B6* ^-/-^ mice after infection (Fig 4E, S1 Table). Furthermore, vascular endothelial *B4galnt2* expressing animals (*RIII* ^+^) exhibited increased *Il-6* expression (*Z* = -1.932, *P* = 0.0528), but decreased LCN-2 production (S1 Table), suggesting a role for vascular *B4galnt2* expression in the host immune response to intestinal infection (Fig 3).

**Fig 5:**
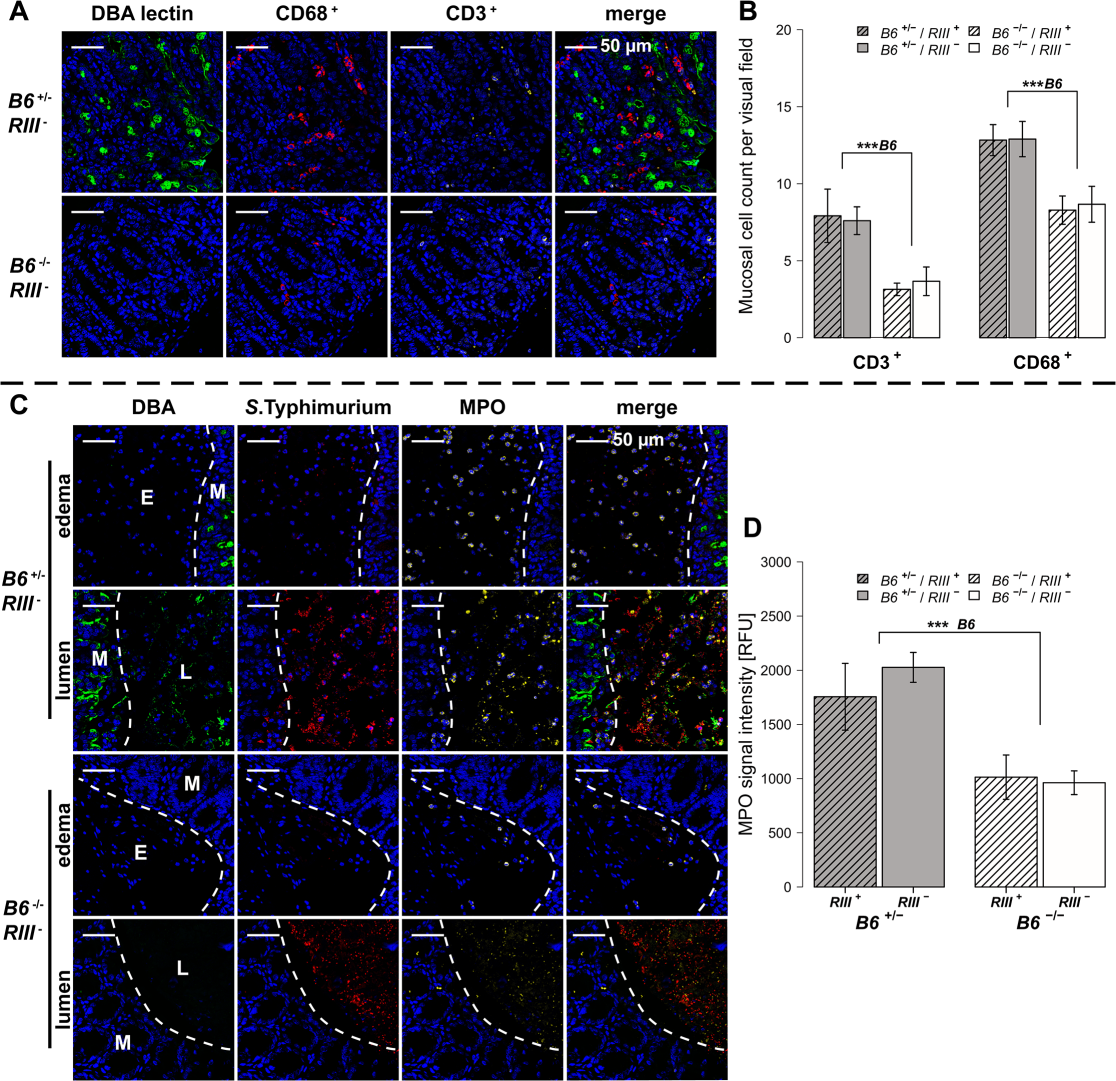
*B4galnt2*-dependent infiltration of immune cells after *S*. Typhimurium infection. (**A** and **B**) Immunofluorescence staining and enumeration of positive cells per vision field showed that *B6* ^+/-^ mice have higher numbers of CD68 (red) and CD3 (white) positive cells in the cecal mucosa 1d p.i. (N = 5–7). Nuclei were stained with DAPI (blue) and *B4galnt2* glycans by using fluorescein labeled DBA (green). (**C**) Myeloperoxidase (MPO) positive cells (white) and *S*. Typhimurium (red) were determined by immunofluorescence staining. (**D**) MPO signal in lumen and edema was quantified and expressed as relative fluorescence units (RFU) (N = 7; Linear model; # *P* < 0.100, * *P* < 0.050, ** *P* < 0.010, *** *P* < 0.001, error bars indicate SEM).

We also analyzed cecal tissue sections for the presence of cells positive for CD68, which is strongly expressed by monocytes and macrophages, and CD3, which is expressed on mature T cells. Immunohistochemical staining and subsequent quantification of cell numbers revealed no difference in cell numbers according to endothelial *B4galnt2* expression (*RIII*), but significantly fewer CD68 ^+^ and CD3 ^+^ cells were observed in the cecal tissues of *B6* ^-/-^ mice (Figs 5A, 5B and S3A and S1 Table) after infection. The presence of neutrophils was further investigated by myeloperoxidase (MPO) staining. In line with our previous results, *B6* ^-/-^ had fewer MPO positive cells in the intestinal mucosa (lumen and edema) compared to *B6* ^+/-^ mice (Figs 4C and S3B) one day p.i., which was further quantified by the relative fluorescence signal intensity (*P* = 0.0001; Fig 4D, S1 Table). Overall, we detected significant differences in the abundance of CD68 ^+^ and CD3 ^+^ cells after infection with respect to the expression of *B4galnt2* in the intestinal epithelium, but almost no differences with respect to vascular endothelial expression.

### Bacterial diversity within and between mice is influenced by intestinal epithelial expression of *B4galnt2*

To examine the effect of *B4galnt2* genotype on the intestinal microbiota in the context of infection, pyrosequencing of the 16S rRNA gene in fecal samples was performed for each individual before and after streptomycin treatment, and after *S*. Typhimurium infection. This resulted in a total of 122,818 sequences, with an average of 998.52 ± 13.49 SD reads per sample after normalization (Good’s coverage of OTUs: 92.46 ± 9.05% SD).

**Fig 6:**
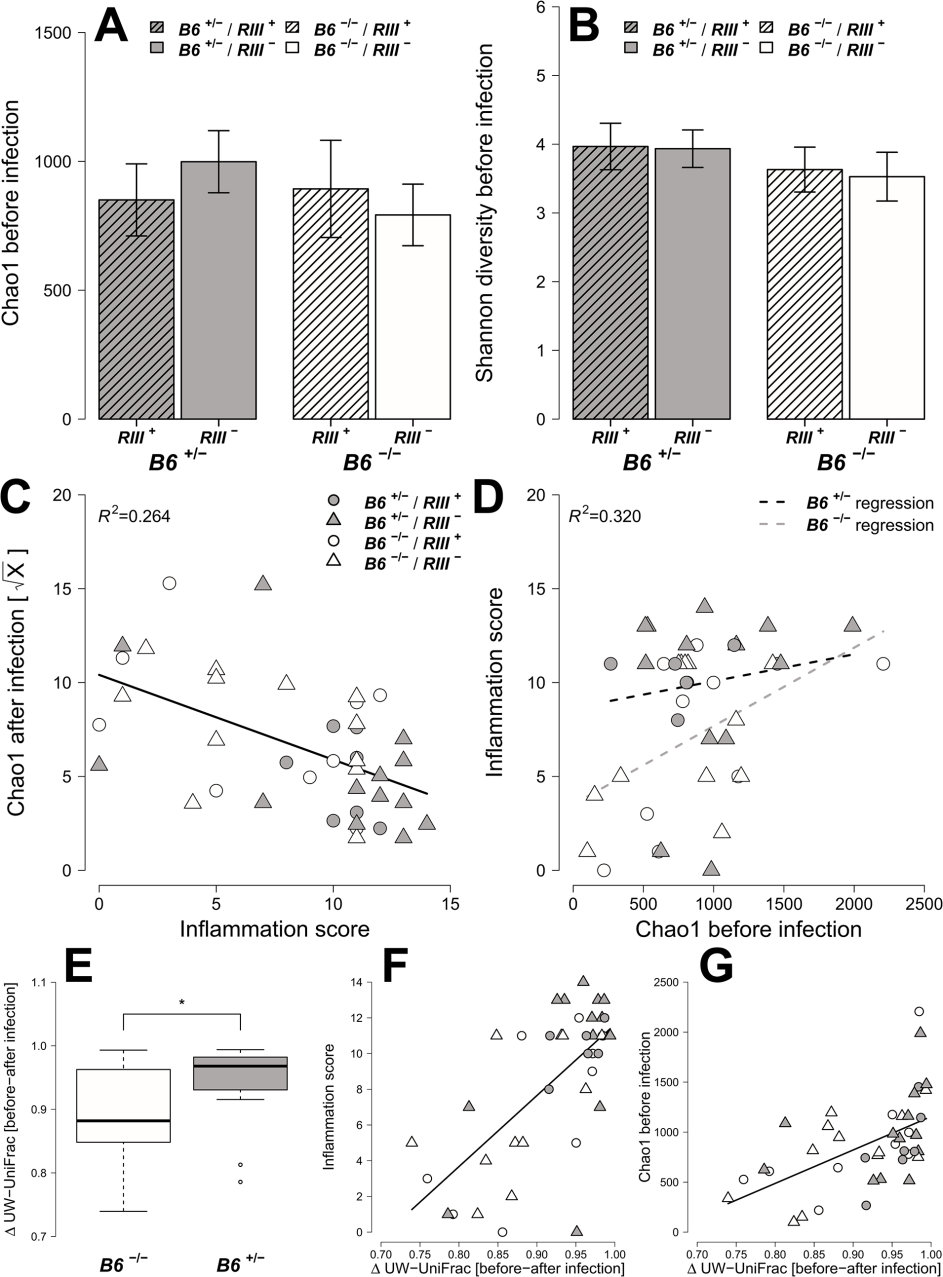
Analysis of microbial alpha diversity among genotypes and their association with intestinal inflammation. Microbial diversity was estimated from 97% species level OTUs and focused on the mean species richness (A; Chao1), and mean abundance based diversity (B; Shannon H), in the untreated animals. (C) The bacterial species richness is (i) decreasing with increasing inflammation (*F*_1,22_ = 14.2123, *P* = 0.0011; LMM), (ii) but highly predictive of inflammation with differences among *B4galnt2* genotypes (D; Chao1 *F*_1,21_ = 9.8274, *P* = 0.005, *B6*: *F*_1,21_ = 9.2976, *P* = 0.0061, see also Table 2). The predictive power of alpha diversity for the outcome of infection is significantly improved by incorporating the *B6* genotype (Chao1: *R* ^*2*^_*LR*_ = 0,320, ΔAIC = -5.936, *LR* = 7.9360, *P*_*LR-Test*_ = 0.0048; Shannon H: *R* ^*2*^_*LR*_ = 0,271, ΔAIC = -6.1811, *LR* = 8.1811, *P*_*LR-Test*_ = 0.0042; NTI: *R* ^*2*^_*LR*_ = 0.2625, ΔAIC = -8.8842, *LR* = 10.8842, *P*_*LR-Test*_ = 0.001). The turnover of bacterial communities (Δ unweighted UniFrac) over the course of the experiment is strongest in animals expressing *B4galnt2* in the epithelium (E; *Z* = -2.3213, *P* = 0.01978; Wilcoxon test via Monte-Carlo resampling), and is highest in animals with strong inflammation (F; *ρ* = 0.5894, *P* = 0.00005; Spearman rank correlation). The community disturbance is also highest in animals with a high species richness before treatment (G; *ρ* = 0.6040, *P* = 0.000042; Spearman rank correlation; # *P* < 0.100, * *P* < 0.050, ** *P* < 0.010, *** *P* < 0.001, error bars indicate SEM; only results of best models are shown and pairwise tests are indicated).

To obtain a detailed picture of the interaction of microbial communities with host factors, we first assessed within-sample (alpha) diversity at multiple complementary levels including species richness (Chao1), distribution (Shannon H), and two phylogenetic measures including Nearest Taxon Distance (NTI) and the Net Relatedness Index (NRI) [41]. Species diversity within and between bacterial communities was strongly influenced by the administration of streptomycin and *S*. Typhimurium (S4 Fig). Prior to streptomycin treatment and infection, the richness and evenness of operational taxonomic units (OTUs) show no significant differences according to *B4galnt2* genotype (Fig 6A and 6B, and Table 1) in concordance with the results of Staubach *et al*. [26]. Phylogenetic clustering among close relatives (*i*.*e*. NTI) is significantly increased in animals with *B4galnt2* expression in the endothelium (*RIII* ^+^), while clustering of large phylogenetic groups (*i*.*e*. NRI) shows no discernable patterns (S5 Fig, Table 1).

**Table 1:** Results of the alpha diversity analyses before and after infection with *S*. Typhimurium (best models after REML fitting).

Time point	Metric	Factor	DF	*F*-value	*P*-value	*R* ^*2*^_*LR*_
before	Shannon H (X^2^)	Intercept	1,22	38.7456	<0.0001	0.0354
treatment		*RIII*	1,22	1.6823	0.2081	
	Chao1	Intercept	1,23	95.9510	<0.0001	0.0737
		Gender	1,13	3.0484	0.1044	
	NRI	Intercept	1,23	50.9385	<0.0001	0.0239
		Gender	1,13	1.1028	0.3128	
	NTI	Intercept	1,22	365.5594	<0.0001	0.1234
		*RIII*	1,22	5.3731	0.0301	
1 d.p.i.	Shannon H	Intercept	1,22	126.3060	<0.0001	0.2538
		Inflammation	1,22	13.7716	0.0012	
	Chao1 (X^1/2^)	Intercept	1,22	101.5123	<0.0001	0.2644
		Inflammation	1,22	14.2123	0.0011	
	NRI	Intercept	1,18	123.7569	<0.0001	0.2019
		*RIII*	1,18	1.9857	0.1758	
		poly(Inflammation)*	2,18	1.3985	0.2725	
		*RIII*:poly(Inflammation)	2,18	2.2966	0.1293	
	NTI	Intercept	1,22	100.8313	<0.0001	0.1184
		Inflammation	1,22	5.3981	0.0298	

* quadratic polynomial fit

After *S*. Typhimurium infection, the number of species and the evenness of their distribution showed a clear decrease with inflammation (Figs 6C and S5C). Phylogenetic clustering of deep branches, on the other hand, is only weakly influenced by genotype and inflammation after *S*. Typhimurium infection (S5E Fig, Table 1), while terminal phylogenetic clustering (NTI) shows a strong negative correlation to inflammation (S5 Fig, Table 1). In addition, the abundance of *S*. Typhimurium detected by 16S rRNA gene sequencing is influenced by *B6-* and *RIII* genotype, especially the low abundance observed in the *RIII* ^+^/*B6* ^-/-^ genotype (S6 Fig), which is consistent with the observations based on colony forming units (Fig 1D; see above).

Next, we attempted to determine which aspects of microbial communities may be associated with infection susceptibility by correlating diversity measurements *prior* to antibiotic treatment to the outcome of infection (inflammation score, *S*. Typhimurium load). Species richness, distribution, and the amount of phylogenetic clustering displayed a significant relationship to the severity of infection outcome, whereby pathology is predicted with relatively high power (Table 2). Furthermore, epithelial *B4galnt2* expression (*i*.*e*. *B6*) significantly increases predictive power (Figs 6D and S7) and may therefore be an important factor modifying the involvement of the microbiota during pathogenesis. Specifically, species loss (ΔChao1) caused by the streptomycin and *S*. Typhimurium infection, which is higher in phylogenetically clustered and species rich communities (ΔChao1~NTI before infection, *ρ* = -0.4216, *P* = 0.006435, ΔChao1~Chao1 before infection, *ρ* = -0.9854, *P* < 2.2 × 10^−16^; Spearman rank correlation) may explain why high species diversity before treatment is correlated to a high inflammatory response (Table 2). Community resistance, measured here as the community turnover (Δunweighted UniFrac) between the pre- and post-infection time points, is higher in *B6* ^-/-^ mice (*i*.*e*. lower Δunweighted UniFrac; Figs 6E, 6F, S8D, S8H and S8L) and shows a strong positive correlation with inflammation and species diversity (Figs 6F, 6G and S8 and S2 Table). Interestingly, the community turnover between the untreated and streptomycin treated communities (before infection) is not associated to the final *Salmonella* load or severity of inflammation. Thus, *B4galnt2* expression in the gut epithelium influences the diversity and resistance of bacterial communities, which in turn is associated with the outcome of infection. Furthermore, these results also underscore the metastable character of highly diverse communities, as was already implied by May in 1972 [42].

**Table 2:** Prediction of inflammatory response by different aspects of alpha diversity (best models after REML fitting).

Factor	*DF*	*F*-Value	*P*-Value	*R* ^*2*^_*LR*_
Intercept	1,21	22.3707	0.0001	0.3200
Chao1	1,21	9.8274	0.0050	
*B6*	1,21	9.2976	0.0061	
Intercept	1,21	19.5089	0.0002	0.2707
Shannon H	1,21	4.4470	0.0471	
*B6*	1,21	10.5759	0.0038	
Intercept	1,21	27.2684	<0.0001	0.2625
NTI	1,21	3.4459	0.0775	
*B6*	1,21	12.1853	0.0022	
Intercept	1,21	27.5336	<0.0001	0.2212
NRI	1,21	1.2906	0.2687	
*B6*	1,21	10.1947	0.0044	
Intercept	1,21	21.8733	0.0001	0.3505
ΔChao1 [before-after *S*. T. infection]	1,21	13.6973	0.0013	
*B6*	1,21	8.8243	0.0073	

**Fig 7:**
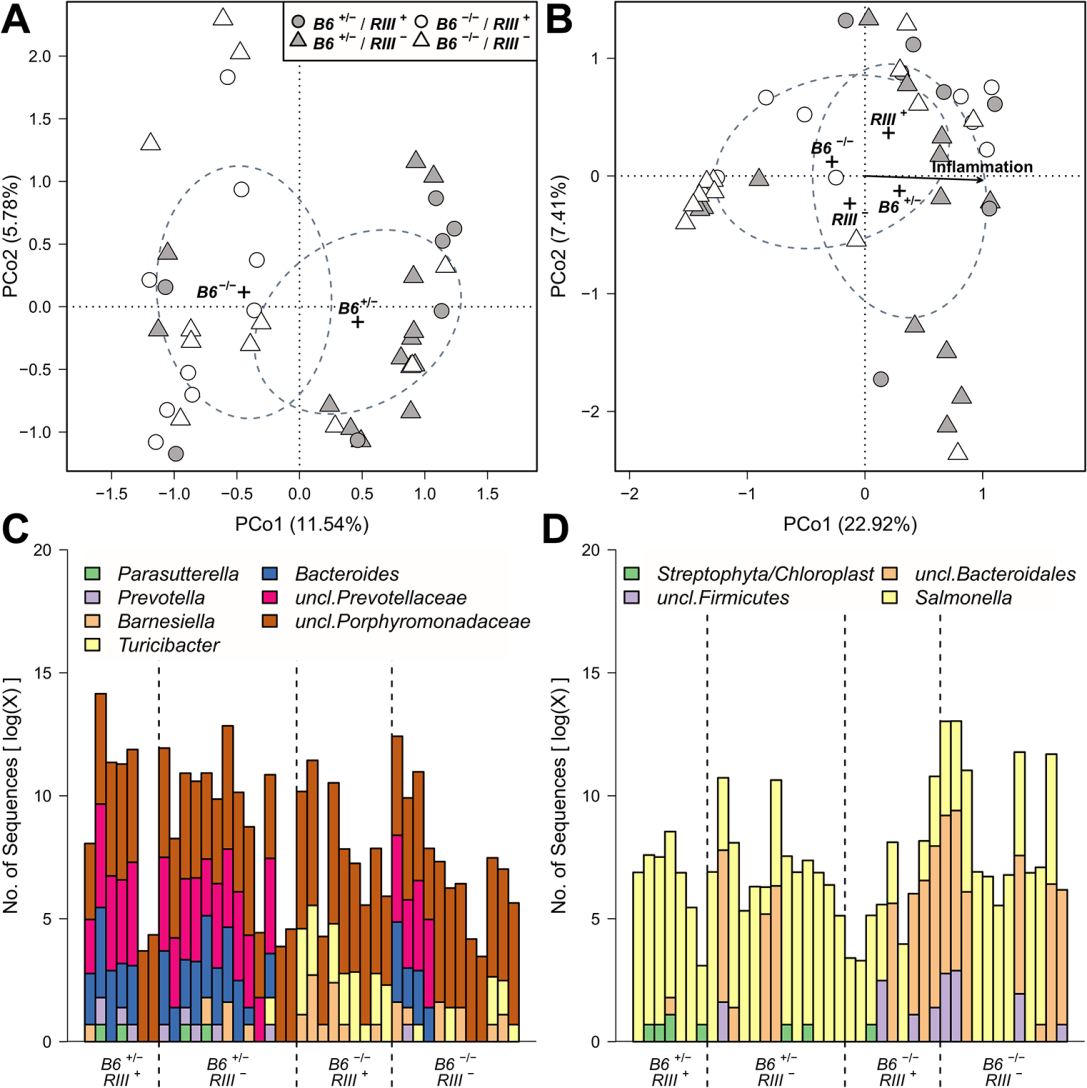
Treatment wise Principle Coordinate Analysis (unweighted UniFrac) of untreated- and *S*. Typhimurium inoculated mice and distribution of indicator bacteria among mice. The significant sample clusters and correlations are shown, displaying a strong influence of epithelial *B4galnt2* expression on the microbial community composition (no treatment (A): *R* ^2^ = 0.1480, *P* = 0.0019; *Salmonella* treatment (B): *B6*: *R* ^2^ = 0.0607, *P* = 0.08669, *RIII*: *R* ^2^ = 0.0781, *P* = 0.040, inflammation: *R* ^2^ = 0.5531, *P*<0.0001). Abundance distribution of indicator genera before (C) and after *S*. Typhimurium infection (D) for *B4galnt2* gut expression among mice.

To infer whether differences *between* the bacterial communities of mice with different *B4galnt2* expression patterns may contribute to susceptibility, we performed beta diversity analyses. Accordingly, diversity between communities was measured based on different characteristics in untreated animals, including OTU- presence/absence (Jaccard/JA),-abundance (Bray-Curtis/BC) and-distribution (Redundancy Analysis/RDA), in addition to the presence/absence- (unweighted UniFrac/UW-UF) and abundance of phylogenetic branches (weighted UniFrac/W-UF). This yielded similar community differences with respect to *B6* genotype in nearly all measures (Figs 7A and S9 and S3 Table) and importantly, confirms the previous findings of Staubach *et al*. 2012 [26] with the current cohort of mice, which were re-derived and housed in a different animal facility. In addition, the bacterial communities among *B6* ^+/-^ animals displayed far less inter-individual variation in their community composition than *B6* ^-/-^ animals (S9 and S10 Figs).

Differences in community structure *after S*. Typhimurium infection were also evaluated and correlated with inflammation score as an additional variable. This showed that differences in communities with respect to *B4galnt2* genotype are also present after infection. Furthermore, the communities changed their species composition with increasing inflammation, which appeared to be most prominent in the microbiota of *B6* ^+/*-*^ animals (RDA: *B6-F*_1,38_ = 3.4908, *P* = 0.0022, inflammation- *F*_1,38_ = 5.0547, *P* = 0.0002, adjusted *R* ^2^ = 0.1406; Figs 7B and S8 and S3 Table). Lastly, the inter-individual distance among *B6* ^-/-^ also remained higher after *S*. Typhimurium infection (S9 and S10 Figs).

### Indicator species and genera characterize the bacterial communities according to intestinal epithelial expression of *B4galnt2*

**Fig 8:**
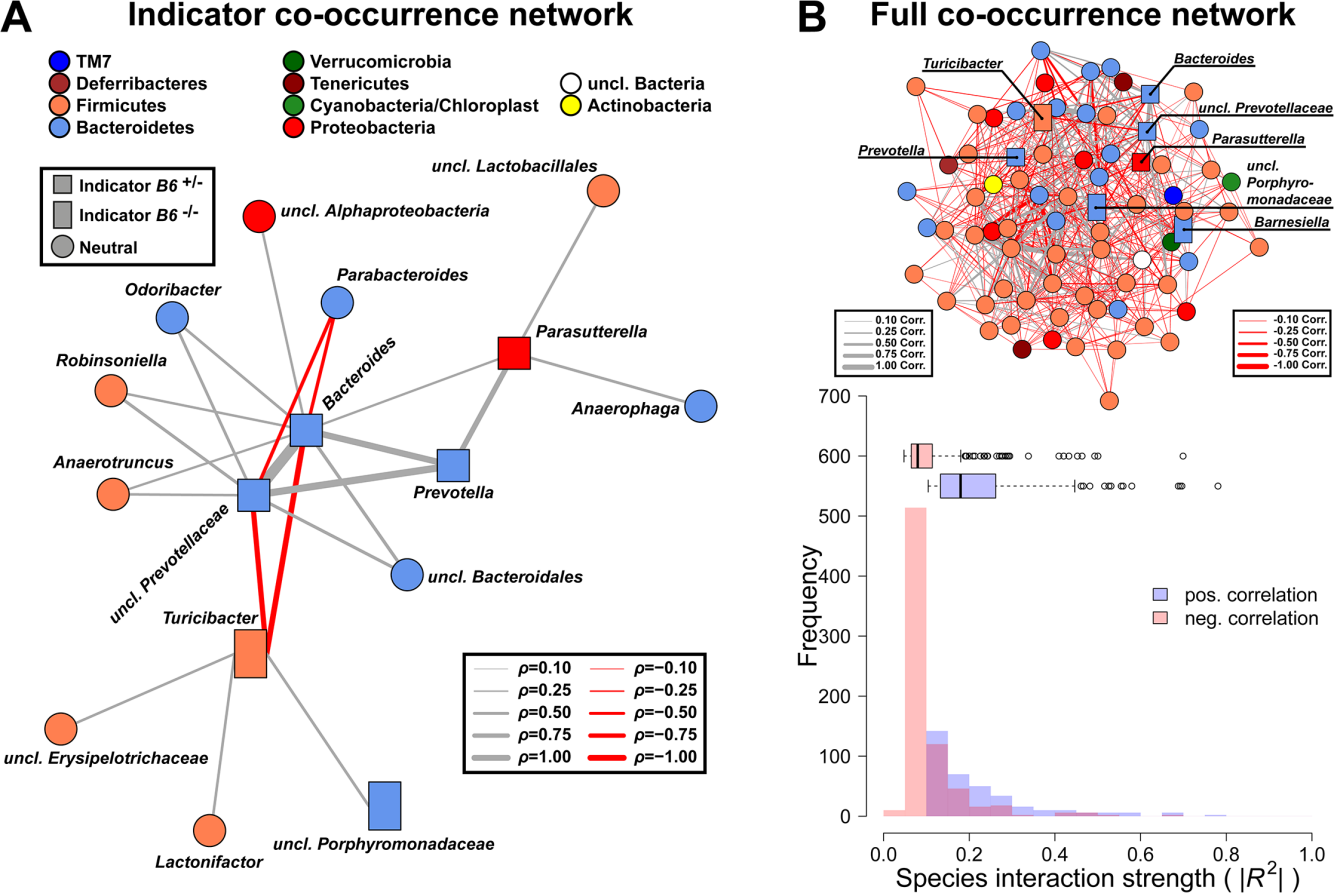
Targeted co-occurrence network analysis of indicator genera and overall network analysis. (**A**) Indicator genera for *B6* genotypes were correlated to abundances of the remaining community members to investigate proximate interactions among indicator genera and the surrounding community (interactions are Spearman correlations see S6 Table; square - *B6* ^+/-^ indicator, rectangle—*B6* ^-/-^ indicator, circle—no indicator/neutral). (**B**) Microbial co-occurrence network based on genera abundances (only significant associations shown), with indicator species highlighted. Microbial communities show significant higher interaction strength among positive interactions (*i*.*e*. potential mutualistic; SPF: *W* = 489396, *P* < 2.20 × 10^−16^; Wilcoxon test). However, the higher frequency of negative weak interactions overall has a stabilizing effect preventing the communities from collapsing (positive/negative interactions = 0.482; # *P* < 0.100, * *P* < 0.050, ** *P* < 0.010, *** *P* < 0.001).

To investigate the drivers of community differentiation between *B4galnt2* genotypes, we employed indicator species analysis. Before treatment and subsequent infection, several genera and species were associated with *B4galnt2* expression (*B6* ^+/*-*^) in the gut, including members of the *Bacteroidales* (*Bacteroides*, *Prevotella*, *Prevotellaceae*) and *Parasutterella* (Proteobacteria), while *Turicibacter* (Firmicutes) and other members of the *Bacteroidales* (*Barnesiella*, *Porphyromonas*, *Porphyromonadaceae*) were indicative of mice lacking *B4galnt2* expression in the gut (*B6* ^-/*-*^; Fig 7C and 7D and S4 and S5 Tables). In addition, *Turicibacter*, *Erysipelotrichaceae*, and *Marvinbryantia* (Firmicutes) are associated to endothelial expression of *B4galnt2* glycans (*RIII* ^+^; Fig 7C and 7D and S4 Table). To further understand the nature of potential interactions among indicator taxa, we performed a targeted correlation network analysis using Spearman rank correlations of the indicator genera to the remaining community members. Interestingly, the genera displaying differential preferences with respect to *B4galnt2* genotype were also negatively correlated with one another, suggesting competitive exclusion mediated by the presence/absence of *B4galnt2* glycans (*Turicibacter*-*Bacteroides*: *ρ* = -0.485, *P* = 0.0013; *Turicibacter*-*uncl*.*Prevotellaceae*: *ρ* = -0.447, *P* = 0.0034). Further, only *Turicibacter*, which is an indicator for the lack of *B4galnt2* expression in the gut, is directly correlated to the indicators of *B6* ^+/-^ genotype while *uncl*. *Porphyromonadaceae* (*B6* ^-/-^ indicator) are only associated to *Turicibacter* abundance (Fig 8A). Through this analysis we additionally found *Parabacteroides* as negatively associated to *Bacteroides* and *Prevotellaceae*, suggesting either competition for *B4galnt2* glycans or a secondary indicator for their absence (Fig 8A and S6 Table). Furthermore, we detected associations of taxa post infection, such as an overabundance of *Salmonella* and *Cyanobacteria* in *B6* ^+/-^, and *uncl*. *Bacteroidales* and *uncl*. Firmicutes in *B6* ^-/-^ mice. Interestingly, we found taxa associated to *B4galnt2* expression in the gut overlapping with a previous study by Staubach *et al*. (2012), such as *Barnesiella* and *Porphyromonadaceae* (S4 and S5 Tables) [26], which further strengthens the evidence for interactions with *B4galnt2* given the independence of these cohorts of mice (see above). Lastly, we explored the dataset for individual taxon associations with inflammation, revealing *Turicibacter* and *Salmonella* to be positively associated to inflammation, potentially benefiting from the inflammatory reactions at the epithelial barrier. Other indicators for the absence of *B4galnt2* glycans like *Parabacteroides* or *Prophyromonadaceae*, however, decline with increasing inflammation (S7 Table). Only the unclassified *Erysipelotrichaceae*, which are secondary indicators for the absence of *B4galnt2* glycans in the epithelium (see Fig 8A and S6 and S7 Tables), are potential probiotic bacteria whose abundance prior to treatment decreases with inflammation (*ρ* = -0.320, *P* = 0.0417). The analysis of the complete co-occurrence network revealed strong dependencies among community members before treatment (Fig 8B). Specifically, we found a higher incidence of weak negative interactions (competition), and a low number of very strong positive interactions (Fig 8B). The co-occurrence network after S. Typhimurium infection shows a comparable distribution of positive and negative interactions, as observed before infection (S11A Fig). Further, it reveals the widespread impact of *Salmonella* (indicator of *B6* ^+/-^) on the microbial community, as its position is highly central and strongly influences several other highly integrated parts of the community (S11B Fig).

### Increased susceptibility of *B6* ^+/*-*^ mice to *S*. Typhimurium triggered inflammation is dependent on microbiota composition

**Fig 9:**
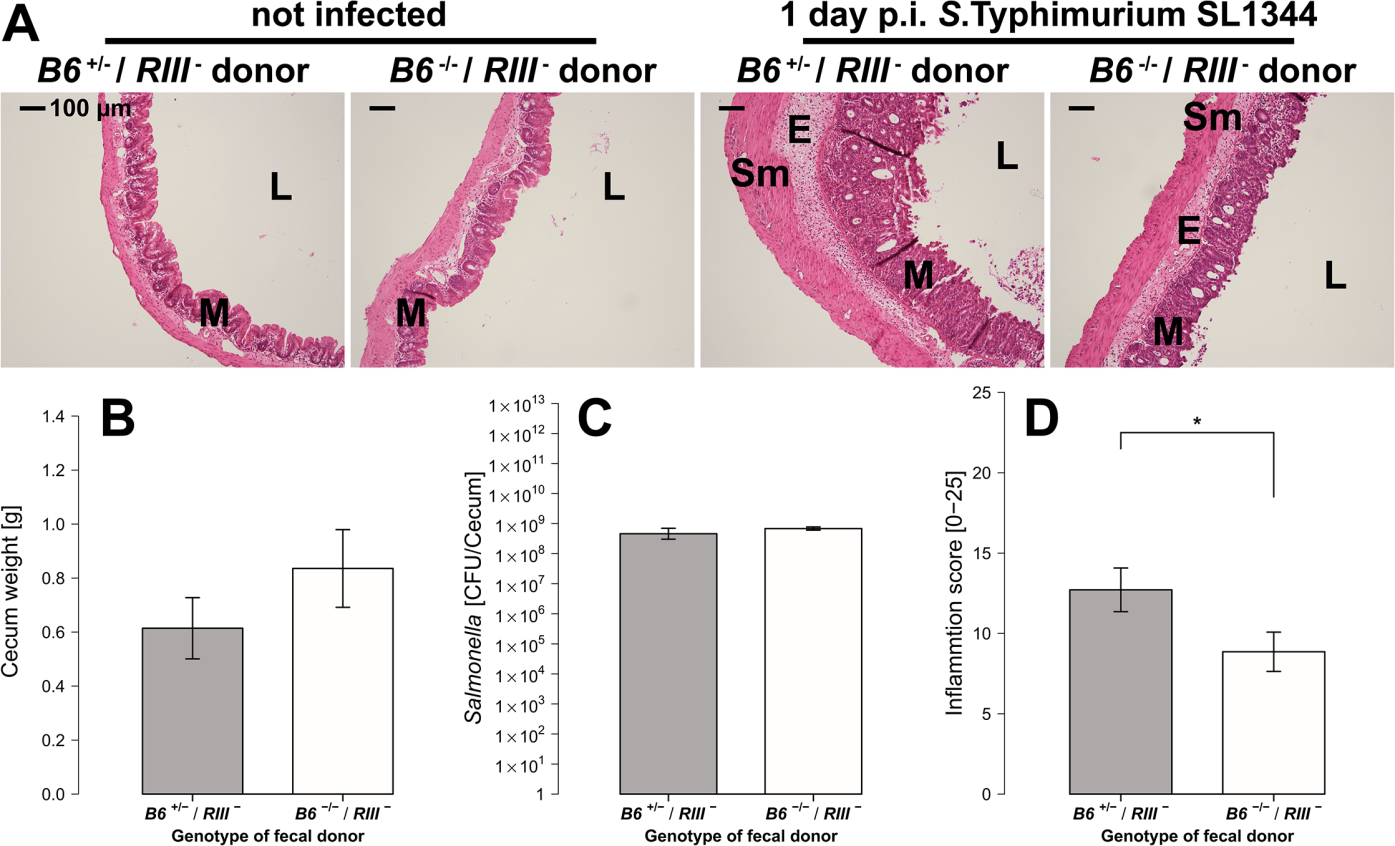
*B4galnt2*-dependent microbiota composition is responsible for enhanced susceptibility to inflammation. (**A**) Representative H&E staining of cecal sections with higher number of luminal cells (L), increased influx of inflammatory cell populations into the mucosa (M) and epithelial cell desquamation and submucosal edema (E) upon infection with *S*. Typhimurium (bar = 100 μm). (**B**) Cecum weight (*Z* = 1.087, *P* = 0.3013, (**C**) and *Salmonella* abundance in the cecum (*Z* = 0.447, *P* = 0.7098) do not differ between donor genotypes (N = 7 infected and N = 3 uninfected controls per donor genotype). (**D**) Histological inflammation is significantly reduced in mice that received a *B6* ^-/-^ microbiome (*Z* = -2.074, *P* = 0.0459; Wilcoxon test via Monte-Carlo resampling, # *P* < 0.100, * *P* < 0.050, ** *P* < 0.010, *** *P* < 0.001, error bars indicate SEM).

In order to determine whether the microbiota composition contributes to the elevated susceptibility of *B6* ^+/-^ mice to inflammation, we transplanted feces from *B6* ^+/-^ and *B6* ^-/-^ donor mice into germfree C57BL/6J (*B6* ^+/+^) recipient mice. 21 days post fecal transplantation, mice were treated with streptomycin and 24 hours later infected with *S*. Typhimurium. Cecum weight and *S*. Typhimurium colonization (CFU count) do not differ significantly between the fecal donor genotypes (Fig 9A, 9B and 9C). However, the extent of tissue inflammation caused by *S*. Typhimurium infection was significantly lower in mice transplanted with microbiota from *B6* ^-/-^ mice due to decreased mucosal damage and decreased submucosal edema (Fig 9A and 9D). These results demonstrate that the differences in microbiota composition from *B6* ^+/-^ and *B6* ^-/-^ mice are responsible for the lower susceptibility of *B6* ^-/-^ mice to *Salmonella* induced inflammation.

## Discussion

Infectious diseases are one of the strongest selective forces on many levels of biological complexity. Over time, a steady cycle of adaptation and counter-adaptation has left molecular traces in the genomes of many organisms including humans [43]. The most prominently affected members are genes associated with the immune system, *e*.*g*. MHC [44], however, others including blood-group-related genes display similar signatures of selection [3, 8, 45–47]. In this study, we investigated intestinal infection as a potential driver of selection at *B4galnt2* observed in the wild by studying the effect of variant tissue-specific expression of *B4galnt2* on host-microbiota interactions and susceptibility to intestinal infection with *Salmonella*. This revealed strong evidence for the influence of *B4galnt2*-specific host glycosylation on microbial community composition and a role in pathogen resistance.

Our experiments revealed less intestinal pathology, lower inflammatory responses, and changes in microbial community structure and composition in animals lacking *B4galnt2* expression in their intestinal epithelium. Host mucosal glycans can directly interact with the microbiota by serving as specific attachment sites or as nutrient sources for some microorganisms. Thus, host mucosal carbohydrates can influence, directly and indirectly, the establishment of overlapping competitive niches, which serve as a barrier against potential pathogens (*i*.*e*. “colonization resistance”) [48, 49]. We found *B4galnt2*-expression-dependent characteristics of the intestinal microbiota, such as species and phylogenetic diversity, which predict the colonization success of *S*. Typhimurium and the severity of the accompanying intestinal inflammation. In our experiment, species-rich and phylogenetically clustered microbial communities appear to be more vulnerable to *Salmonella* infection, and ultimately inflammation. Before the seminal works of May and others [42, 50, 51], high diversity habitats were synonymous with high stability and productivity [52, 53]. However, the diversity-stability debate remains unresolved [54–56]. High diversity only has a stabilizing effect if reactions of community members are asynchronous, which balances the reduction of one species by the complementary increase of other community members [57–59]. This “portfolio”- [60] or “insurance” effect [61] dampens perturbations by a release of inter-species competition, or by differential susceptibility to the environmental stressors [54]. Diverse communities also exhibit an intrinsically higher tendency of community change, as a large number of species (especially rare species) are prone to becoming lost through environmental perturbation and stochastic events due to their limited relative abundance [62]. This has been observed in grassland communities, where compositional instability increases with community diversity [63]. Thus, the comparably high number of strong positive interactions in the bacterial communities of this study (see Fig 8B) may therefore explain the tendency of exacerbated species loss and inflammation after disturbance, as the stabilizing effects of competitive release are lower [57, 60, 64, 65]. Furthermore, evolutionary relatedness among community members has a strong influence on community reactions and productivity. Closely related species (*e*.*g*. phylogenetically clustered) presumably overlap in their niches and functional capacities [66, 67] and react in similar ways to environmental stressors, which dampens the insurance effect (*i*.*e*. “negative insurance effect”) as observed in the investigated microbial communities [68, 69].

Antibiotic treatment usually has long lasting effects, but previous studies show that a certain degree of resilience occurs through short-term repopulation of dormant bacteria [49, 70]. The disturbance in microbial communities appears to be buffered in mice not expressing *B4galnt2* glycans in the epithelium, possibly by conferring “colonization resistance” via a higher potential to compete with invading *Salmonella* and by dampening the effects of community disturbance [67, 71, 72]. Thus, in the context of a diminished and disturbed microbial community after streptomycin treatment [73], it is likely that the more resilient/resistant communities in *B6* ^-/-^ mice maintain a greater potential for rapid recovery [48, 70, 74]. We further postulate that *B4galnt2* genotype-dependent host-microbe interactions modulate the host’s immune response, contributing to less severe pathology and increased pathogen clearance in mice lacking intestinal epithelial *B4galnt2* expression.

Commensal gut bacteria benefit from the intestinal mucus and its diverse glycan residues, as they offer a complex repertoire of binding sites and carbohydrate sources independent of the host diet [1, 3, 75, 76]. The indicator species identified for mucosal *B4galnt2* expression, *Prevotella* and *Bacteroides*, are known to digest and bind a large spectrum of glycans [77]. These bacteria of high metabolic potential show signs of niche competition with the genus *Turicibacter*, an indicator for *B4galnt2*-deficient mice. *Turicibacter*, *e*.*g*. *Turicibacter sanguinis*, is a known member of the human and murine gut microbiome, but can only utilize a narrow range of carbohydrates [78]. As suggested by Dimitriu *et al*. (2013) [79], the trade-off between low metabolic capacity and competitive abilities [78, 80] with the potential for fast colonization might explain the association of *Turicibacter* with *B6* ^-/-^ mice and the co-increase with *S*. Typhimurium [81–83]. It was also suggested that *Turicibacter* possesses immune modulatory characteristics (increasing iNK T cell, and marginal zone B cell abundance [84]), and may thus help to lower the susceptibility to gut inflammation in *B6* ^-/-^ compared to *B6* ^+/*-*^ mice in the face of equivalent *Salmonella* burdens [79]. However, *Turicibacter* could also benefit from existing tissue inflammation, as several genomic features such as laminin, internalin, or a collagen binding pilus allow this genus to act as an opportunistic pathogen, and thus explain its association with tissue inflammation [78, 80]. Similarly, *Barnesiella* shows repeated association to the absence of *B4galnt2* glycans [26]. This genus has the potential to counteract inflammatory responses and thus appears to play a central role in the gut microbiome [85].

Co-staining of MUC2 and DBA lectin demonstrated a partial co-localization in goblet cells, suggesting that MUC2 is glycosylated by B4galnt2 in agreement with previously published data [31]. However, *B4galnt2* glycans were also detectable in the cecal mucosa of *Muc2*-deficient mice (Figs 2A and S2A), indicating additional intestinal targets of B4GALNT2 glycosylation. Other glycosylation targets for B4galnt2 are Sd(a)/Cad antigens, which have been shown to be present in colonic mucins [34, 36], glycolipids and glycoproteins [32, 33, 35, 86]. Intestinal mucin glycans, including blood group α-1,2 fucosylated receptors, have been proposed as attachment sites for *Salmonella* [87, 88], but *Salmonella* does not appear to directly bind *B4galnt2*-GalNAc residues *in vitro* [4]. The glycan profile may also change in animals not expressing *B4galnt2* in addition to the lack of β1,4-GalNac residues/Sd(a), whereby the increase or decrease of other residues may offer new nutrient sources or attachment sites for bacteria or immune cells [35, 89]. Nevertheless, we found slightly increased invasion into epithelial cells *in vivo* and *in vitro* when *B4galnt2* is expressed. However, our fecal transfer experiments demonstrate that the altered bacterial community of *B6* ^-/-^ mice confers resistance towards *Salmonella* induced inflammation. Thus, it is likely that indirect mechanisms, such as the microbial community and its capability of glycan liberation, subsequent changes in nutrient or microbe abundances [90] and the type of interactions [72], are responsible for the higher susceptibility of mice expressing *B4galnt2* in the intestinal epithelium to *S*. Typhimurium infection.

Our study reveals an increased production of pro-inflammatory mediators, higher numbers of immune/inflammatory cells, and more severe colitis after *S*. Typhimurium infection in the ceca of mice expressing *B4galnt2* in the intestinal epithelium. Although endothelial *B4galnt2* expression did not impact the development of colitis as judged by histology, *RIII* ^+^ mice had lower pathogen burden in the cecum and lower levels of *Mcp-1* and LCN-2 compared to *RIII* ^-^ mice, supporting a role for vascular *B4galnt2* in host immune defense in the face of intestinal pathogens. Functionally, carbohydrate differentiation antigens play an important role in the homing and differentiation of intraepithelial lymphocytes in the small intestine, indicating a plausible phenotype that may result from the expression of *B4galnt2* in endothelial cells [91–94]. The recruitment of neutrophils and CD3 ^+^ cells [35], as well as leukocyte infiltration, were reported to be influenced through the glycosylation of selectin receptors [95] and could be associated with the elimination of carbohydrate ligands for selectins. *B4galnt2* expression in gastrointestinal cancers has been shown to reduce metastatic dissemination, adding to the role of the Sd(a) antigen in cell motility [96, 97]. Further studies focusing on the role of endothelial *B4galnt2* expression are needed to understand the impact of *B4galnt2*-GalNAc residues in host immune responses and its potential role for homing of immune cells to the intestine.

In summary, we demonstrate that different patterns of tissue-specific *B4galnt2* expression not only influence intestinal microbial communities, but also change host susceptibility and immunological responses to *S*. Typhimurium infection [45, 98]. Thus, a complex scenario including *B4galnt2-*dependent changes in microbial communities, vascular immune phenotypes, bleeding tendencies and susceptibility to intestinal infections likely contributes to the maintenance of variation at *B4galnt2* in wild mouse populations.

## Material and Methods

### Animal models of variant B4galnt2 tissue-specific expression

All genetically engineered mouse lines used in the study were backcrossed >20 generations to a C57BL/6J background prior to breeding of the experimental animals. C57BL/6J (*B6* ^+/+^) mice were purchased from The Jackson Laboratory. Mice heterozygous for the *B4galnt2* knock-out allele (*B6* ^+/-^) [23] and RIIIS/J-*B4galnt2* BAC transgenic (*RIII* ^+^) mice which exhibit the *Mvwf1* phenotype [21] were re-derived at the University Clinic Eppendorf, Hamburg, Germany. Intercross of *B6* ^+/-^ × *B6* ^+/-^ *RIII* ^+^ generated heterozygous *B6* ^+/-^/*RIII* ^+^, *B6* ^-/-^/*RIII* ^+^, *B6* ^+/-^/*RIII* ^-^ and *B6* ^-/-^/*RIII* ^-^ offspring, which were raised and housed together as littermates under specific pathogen-free conditions in individually ventilated cages at the animal facility of the University of Kiel, Germany. Standard chow (ssniff, Soest, Germany) and water were provided *ad libitum*. Germ-free C57BL/6J mice were produced at the gnotobiotic facility of the Hannover Medical School. Experiments were conducted in the animal facility of the Leibniz Research Center Borstel, Germany and at the animal facility of University Hospital Schleswig-Holstein Kiel.

### Ethics statement

All experiments were conducted consistent with the ethical requirements of the Animal Care Committee of the Ministry of Energy, Agriculture, the Environment and Rural Areas of Schleswig-Holstein, Germany and in direct accordance with the German Animal Protection Law. The protocols were approved by the Ministry of Energy, Agriculture, the Environment and Rural Areas of Schleswig-Holstein, Germany (Protocol: V312-72241.123–3 and V312-7224.123–3).

### Salmonella infection of mice

Streptomycin (20mg per mouse) (Sigma-Aldrich, Hamburg, Germany) was given by oral gavage to mice aged 10–14 weeks. 24 hours after antibiotic administration, mice were infected with either *S*. Typhimurium SL1344 (acute infection; [28]) or the attenuated *S*. Typhimurium Δ*aroA* (chronic infection; [29]) at a dose of 3 × 10^6^ bacteria in 100 μL HEPES buffer (100 mM, pH 8.0; PAA, Cölbe, Germany). Control mice (mock-infection) were given 100 μL HEPES buffer. Bacterial loads were determined by plating serial dilutions of homogenized organs on Luria Bertani agar (Roth, Karlsruhe, Germany) containing streptomycin (100 μg/mL).

### siRNA knockdown and tissue culture infections

Mouse intestinal epithelial Mode-K cells were grown in DMEM supplemented with 5% fetal bovine serum (Biochrom, Berlin, Germany) and 1% HEPES (GE Healthcare, Frankfurt, Germany). For the siRNA knockdown of *B4galnt2* 1 × 10^5^ cells per well were seeded in a 24 well plate containing 10nM siRNA and lipofectamine (Life Technologies, Darmstadt, Germany) according to manufacturer’s instructions for reverse transfection. As a negative control cells were treated with scrambled siRNA. 24h post transfection cells were infected with an MOI 50 of wildtype *S*. Typhimurium grown to late-logarithmic phase. 30 min p.i., cells were washed and extracellular bacteria were killed by addition of medium containing gentamicin (100 μg/ml). Cells were lysed at various timepoints (30 min, 1 h and 4 h) and the number of adherent and invaded bacteria was determined by plating serial dilutions.

### Fluorescence in situ hybridization (FISH) staining

Cecal tissues were fixed in Carnoy’s fixative overnight, embedded in paraffin, and then cut in 5 μm sections on glass slides. Sections were deparaffinized and incubated with a Texas red-conjugated EUB338 general bacterial probe (5’-GCTGCCTCCCGTAGGAGT-3’) and an AlexaFluor 488 conjugated Gam42a probe (5’-GCCTTCCCACATCGTTT-3’) that recognizes bacteria that belong to the γ-Proteobacteria class (37°C, O/N, dark). Tissue samples were washed with hybridization buffer (0.9 M NaCl, 0.1 M Tris pH 7.2, 0.1% SDS). This step was repeated with FISH Washing Buffer (0.9 M NaCl, 0.1 M Tris pH 7.2) with gentle shaking for 15 minutes. Sections were washed with water and mounted using Prolong GOLD with DAPI (Molecular Probes) and imaged using an AxioImager microscope equipped with an AxioCam HRm camera operating through AxioVision software. High power field (HPF) (630X) was used for enumerating intracellular and extracellular *S*. Typhimurium.

### Staining of acidic mucus and mucus thickness

Carnoy’s-fixed paraffin-embedded tissues were sectioned (5 μm), deparaffinized, and stained with 1% Alcian Blue (Sigma-Aldrich, Hamburg, Germany) solution (in 1% acetic acid) for 10 min, counterstained in nuclear fast red solution (1%), dehydrated, and mounted for examination. Photographs were taken at an original magnification of 100x and mucus thickness was measured at six random locations per section using NIS-Element Software (Nikon, Dusseldorf, Germany).

### Fecal transplantation experiments

Fresh feces from *B6* ^+/*-*^ or *B6* ^-/-^ mice was sampled and immediately homogenized (1:10 w/v) in transfer buffer (sterile phosphate buffered saline containing 0.05% cysteine HCl (Sigma-Aldrich)). After centrifugation, the supernatant was collected and 200 μL were orally gavaged into germ-free adult C57BL/6J recipient mice. 21 days post transplantation mice were treated with streptomycin and 24 hours later infected with *S*. Typhimurium.

### Histopathological analysis

Tissues were fixed in 10% neutral buffered formalin overnight and embedded in paraffin. 5 μm sections were deparaffinized and stained with haematoxylin and eosine (H&E). Histological scores in the ceca of infected mice were determined as previously described [30]. Briefly, pathological changes were assessed by evaluating various parameters such as presence of luminal cells, infiltrating immune cells, crypt abscesses and the formation of edema in the respective layer of the intestinal bowel wall including the surface epithelium, mucosa and submucosa.

### Immunohistochemistry

Formalin fixed tissue sections (5 μm) were deparaffinized and rehydrated. After antigen retrieval with 10 mM sodium citrate buffer (pH 6.0) and blocking with 2% normal goat serum, specimens were incubated with antibodies specific for *S*. Typhimurium (Clone B395M, Dunn Laboratories, Asbach, Germany), CD3 (Abcam, Cambridge, UK), CD68 (Abcam, Cambridge, UK), myeloperoxidase (MPO) (Thermo Fisher Scientific, Schwerte, Germany), and MUC2 (Santa Cruz, Dallas, TX, USA) followed by fluorescently labeled secondary antibodies (Molecular Probes, Invitrogen, Carlsbad, CA, USA) or with fluorescently labelled DBA (dolichus biflorus agglutinin) and WGA (wheat germ agglutinin) lectins (Vector laboratories, Burlingame, CA, USA). Counterstaining of nuclei was performed using 4,6-Diamidin-2-phenylindol (DAPI) (Invitrogen, Carlsbad, CA, USA). Images were obtained using a Leica SP5 confocal microscope (Leica, Wetzlar, Germany).

### Lipocalin-2 enzyme-linked immunosorbent assay (ELISA)

Lipocalin-2 concentrations in the supernatant of tissue homogenates were determined with a mouse specific ELISA Development Kit by R&D Systems (R&D Systems, Wiesbaden, Germany) according to the manufacturer’s instructions.

### Real-time quantitative polymerase chain reaction (RT-qPCR)

RNA was extracted from cecal tips by using the High Pure RNA Tissue Kit (Roche Diagnostics, Mannheim, Germany) and reverse transcription was conducted with the Transcriptor High Fidelity cDNA Synthesis Kit (Roche Diagnostics, Mannheim, Germany) according to the manufacturer’s instructions. RT-qPCR was performed with Quantitect SYBR-Green Mastermix (QIAGEN, Hilden, Germany) for the following genes: *Ifn-γ*, fw TCAAGTGGCATAGATGTGGAAGAA, rev TGGCTCTGCAGGATTTTCATG; *Tnf-α*,fw CCACCACGCTCTTCTGTCTAC, rev AGGGTCTGGGCCATAGAACT; *Il-6*, fw GAGGATACCACTCCCAACAGACC, rev AAGTGCATCATCGTTGTTCATACA; *Mcp-1*, fw CCTGCTGTTCACAGTTGCC, rev ATTGGGATCATCTTGCTGGT; *B4galnt2*, fw TGGCAAGTCCTACCATGAGG, rev GTCTGCAGAAGTGGCTGGA*; Gapdh*, fw ATTGTCAGCAATGCATCCTG, rev ATGGACTGTGGTCATGAGCC; *Hprt*, fw AGTGTTGGATACAGGCCAGAC, rev CGTGATTCAAATCCCTGAAGT. Relative gene expression was calculated using geNORM and the 2^-∆∆Ct^ method, with *Gapdh* and *Hprt* as housekeeping genes [99].

### DNA extraction and 16S rRNA gene sequencing

DNA was extracted from fecal samples (stored at -80°C) using the PowerSoil DNA Isolation Kit (MO Bio Laboratories, Carlsbad, CA) following the manufacturer’s protocol. The 16S rRNA gene was amplified using barcoded primers flanking the V1 and V2 hypervariable regions (27F-338R) and were sequenced following the methods describe in Rausch et al. 2011 [100].

### Sequence processing and quality control

Raw sequences were trimmed by mothur 1.31.2 requiring no ambiguous bases, a mean quality score within a window of 50 base pairs of ≥ 35 and a minimum length of 200 nucleotides for the coupled V1-V2 region [101]. Chimeric sequences were determined using USEARCH 4.25 (database informed UCHIME algorithm) [102]. Sequences were confirmed as bacterial using the RDP classifier with ≥ 60% bootstrap threshold [103]. For all downstream analyses of diversity and habitat association, we took a random subset of 1000 sequences per sample to normalize the read distribution (Good’s Coverage; no treatment: 85.67 ± 6.61% SD; Streptomycin: 97.38 ± 3.13% SD; *S*. Typhimurium infection: 98.36 ± 1.74% SD). These sequences were aligned to the curated SILVA seed database using the NAST alignment procedure as implemented in mothur and subsequently OTU binning was carried out via average distance clustering [104]. Phylogenetic tree construction on representative OTU sequences (average distant sequence of the OTU) was done by FastTree 2.1 using the CAT substitution model with gamma correction [105]. Raw sequence data can be accessed online under the accession number PRJEB5269 at the European Nucleotide Archive.

### Statistical analysis

Species diversity indices (Chao1 species richness, Shannon-Weaver index), as well as the phylogenetic distance at the tips of the phylogenetic tree (Nearest Taxon Index, NTI) and its deep branches (Net Relatedness Index, NRI) were calculated in R [106–108]. The phylogenetic measures of beta diversity (unweighted- and weighted UniFrac) and metrics based on shared OTU presence (Jaccard) or abundance (Bray-Curtis) were calculated in “vegan” [109–111]. Statistical analysis of community composition based on different beta diversity metrics was performed with Principal Coordinate Analysis (PCoA) and non-parametric multivariate analysis of variance and multivariate dispersion as implemented in the “vegan” package for R with 10^5^ permutations. For constrained ordination (Redundancy Analysis) the OTU table was Hellinger-transformed and RDA was carried out following Legendre and Legendre [112]. Significance of factors and axes was ascertained using a permutative ANOVA approach (5000 permutations). Linear mixed models (LMM, cage as random factor) were applied to alpha diversity measures and optimized with model selection by AIC criterion, normality of model residuals and refitting of the final model under Restricted Maximum Likelihood (REML) [113]. The *R* ^*2*^_LR_ values of the final mixed model were calculated using the MuMIN package for R [114, 115]. Lipocalin-2 levels, fluorescence signals, inflammation scores, CFU counts, and cecum weights were analyzed in a linear model framework with parameter selection to minimize the AIC value and no significant reduction of fit. For the comparison of expression values among genotypes we employed a Wilcoxon test with Monte-Carlo resampling [116]. *Salmonella* counts (Gam24a ^+^ cells) in Mode-K cell cultures were analyzed using an LMM with the independent rounds of experiments as random factor to incorporate experimental variation. Indicator species analysis was based on 10^5^ permutations using the indicator value to assess the association for each taxon [117]. All *P*-values of the genera and OTU associations were adjusted by the Benjamini-Hochberg procedure. Taxon co-occurence networks were calculated by SPARCC based on 10^5^ permutations and significant associations (*P* < 0.05) were included in the network construction [118].

## Supporting Information

S1 Fig Inflammation after chronic infection with *S*. Typhimurium ∆*aroA*. (**A**) We find no difference between mice differing in *B4galnt2* expression in histological inflammation (*Z* = 0.447, *P* = 1.000), *Salmonella* load (**B**; *Z* = -0.747, *P* = 0.5658) and (**C**) cecum weight (*Z* = 0.490, *P* = 0.7311) (Wilcoxon test via Monte-Carlo resampling; # *P* < 0.100, * *P* < 0.050, ** *P* < 0.010, *** *P* < 0.001).
(TIF)

S2 Fig *B4galnt2* glycosylation dynamics in the intestinal mucosa (addition to Fig 2A and 2E). (**A**) Mucin-2 (MUC2) and *B4galnt2* glycan residues (GalNAc) were stained with fluorescein labeled DBA in formalin fixed cecal tissue sections (Sm-submucosa, M-mucosa, L-lumen). (**B**) *B4galnt2* glycan residues (GalNAc) were stained with fluorescein labeled DBA in formalin fixed cecal tissue sections before and 1 days p.i. with *S*. Typhimurium. GlcNAc residues were stained with Alexa633 labeled Wheat Germ Agglutinin (WGA).
(TIF)

S3 Fig *B4galnt2*-dependent infiltration of immune cells after *S*. Typhimurium infection (addition to Fig 5A and 5C). (**A**) Immunofluorescence staining and enumeration of positive cells per vision field showed that *B6* ^+/-^ mice have higher numbers of CD68 ^+^ and CD3 ^+^ cells in the cecal mucosa 1d p.i. (N = 5–7; E-edema, M-mucosa, L-lumen). Nuclei were counterstained with DAPI and *B4galnt2* glycans by using fluorescein labeled DBA. (**B**) Myeloperoxidase (MPO) positive cells and *S*. Typhimurium were determined by immunofluorescence staining in formalin fixed cecal sections (5 μm).
(TIF)

S4 Fig Analyses of microbial alpha diversity and beta diversity among treatments. Microbial diversity was estimated from 97% species level OTUs and focused on species richness (**A**; Chao1: *χ* ^*2*^ = 78.940, *P*<2.2 × 10^−16^; Kruskal-Wallis test), species distribution (**B**; Shannon H: *χ* ^*2*^ = 65.997, *P* = 4.666 × 10^−15^; Kruskal-Wallis test), and distant and close phylogenetic relatedness (C; NRI: *χ* ^*2*^ = 6.4166, *P* = 0.04043; **D**; NTI: *χ* ^*2*^ = 50.4593, *P* = 1.104 × 10^−11^; Kruskal-Wallis test). Community changes among treatments were measured by the Jaccard distance (**E**; *adonis*: *F*_2,120_ = 9.577, *R* ^*2*^ = 0.13765, *P*<0.0001), Bray-Curtis (**F**; *adonis*: *F*_2,120_ = 12.055, *R* ^*2*^ = 0.1673, *P*<0.0001), UW-UF (**G**; *adonis*: *F*_2,120_ = 13.932, *R* ^*2*^ = 0.18845, *P*<0.0001), and W-UF (**H**; *adonis*: *F*_2,120_ = 20.615, *R* ^*2*^ = 0.25572, *P*<0.0001). Within treatment community variability (**I-L**) was also strongly influenced by the treatment regime (J- *F*_2,120_ = 5.5668, *P* = 0.0054; BC- *F*_2,120_ = 9.1942, *P* = 0.0004; W-UF: *F*_2,120_ = 11.832, *P*<0.0001; UW-UF: *F*_2,120_ = 1.7496, *P* = 0.1804)
(TIF)

S5 Fig Analysis of microbial alpha diversity among genotypes and their influence on intestinal inflammation. Microbial diversity was estimated from 97% species level OTUs and focused on species distribution (Shannon H: C), and close and distant phylogenetic relatedness (NTI: **A**, **D**; NRI: **B**, **E**), in the untreated state (**A**, **B**) and 1 day post infection with *S*. Typhimurium (**C-E**; Table 1 for the respective statistics).
(TIF)

S6 Fig Salmonella abundance among *B4galnt2* genotypes based on sequence abundance. *Salmonella* abundance significantly differed between *B6* and *RIII* genotypes (*B6*: *F*_1,20_ = 5.32081, *P* = 0.0319; *RIII*: *F*_1,20_ = 6.91949, *P* = 0.0160; *B6/RIII*: *F*_1,20_ = 2.74565, *P* = 0.1131, *R* ^2^_LR_ = 0.28114; LMM) with the lowest abundance in *RIII* ^+^/*B6* ^-/-^ animals (Tukey pairwise comparisons: *RIII* ^+^/*B6* ^-/—^ *RIII* ^-^/*B6* ^-/-^: *Z* = -3.102, *P* = 0.00979; *RIII* ^+^/*B6* ^-/—^ *RIII* ^-^/*B6* ^+/-^: *Z* = -3.430, *P* = 0.00341; *RIII* ^+^/*B6* ^+/—^ *RIII* ^+^/*B6* ^-/-^: *Z* = 2.582, *P* = 0.04698)
(TIF)

S7 Fig Prediction of infection outcome by alpha diversity. The severity of histological inflammation was significantly predictable by the change of species richness inflicted by *S*. Typhimurium infection and streptomycin treatment (**A**, ΔChao1), by the eveness of species distribution before infection (**B**, Shannon H), and clusteredness of closely related phylogenetic groups before infection (**C**, NTI). Phylogenetic clustering of distantly related species before infecation shows no significant association to the resulting inflammation (**D**, NRI, see Table 2).
(TIF)

S8 Fig Analyses of community disturbance. The community distances between animals before and after treatment were used as a measure of community disturbance, considering (**A-D**) species composition/Jaccard, (**E-H**) species abundance/Bray-Curtis, and (**I-L**) phylogenetic composition/weighted UniFrac. This disturbance signifies an increased species turnover (higher distance) in animals with a diverse microbial community measured in different ways, considering species number, distribution and phylogenetic relatedness (*e*.*g*. Chao1 (**A**, **E**, **I**), Nearest Taxon Index (**B**, **F**, **J**); see also S2 Table). Community turnover also correlates strongly with severity of inflammation, and increased *Salmonella* load (see S2 Table). Furthermore animals lacking epithelial *B4galnt2* expression have on average less disturbance/higher resilience than mice with gut epithelial expression (**D**: Δ Jaccard: *Z* = -2.2731, *P* = 0.02311; **H**: Δ Bray-Curtis: *Z* = -2.2998, *P* = 0.0205; **L**: Δ W-UniFrac: *Z* = -1.6171, *P* = 0.1090; Wilcoxon test via Monte-Carlo resampling; see also Fig 7).
(TIF)

S9 Fig Principal Coordinate Analyses of different beta diversity measures. PCoAs of phylogenetically informed (**A**, **B**) and species based (**C**-**F**) metrics of beta diversity, that show clustering of microbial communities by epithelial *B4galnt2* expression (**C**: *R* ^2^ = 0.1478, *P* = 0.0011; E: *R* ^2^ = 0.1373, *P* = 0.0020) and sex (**A**: *R* ^2^ = 0.0884, *P* = 0.0260) before any treatment. After *S*. Typhimurium infection the community structures show strong and consistent correlation to histological inflammation (**B**: *R* ^2^ = 0.5054, *P*<0.0001; **D**: *R* ^2^ = 0.3167, *P* = 0.0006; F: *R* ^2^ = 0.4935, *P* = 0.0002) and significant discrimination among epithelial and endothelial *B4galnt2* expression patterns (**B**: *B6*-*R* ^2^ = 0.1272, *P* = 0.005199; F: *B6*-*R* ^2^ = 0.0951, *P* = 0.01430).
(TIF)

S10 Fig Community variability between genotypes. Comparison of bacterial community distances (beta diversity) between animals with and without epithelial *B4Galnt2* expression, before and after *S*. Typhimurium infection (not infected- Jaccard: *F*_1,39_ = 4.1584, *P* = 0.04779; Bray-Curtis: *F*_1,39_ = 3.961, *P* = 0.05379, UW-UF: *F*_1,39_ = 5.414, *P* = 0.0246; W-UF: *F*_1,39_ = 1.235, *P* = 0.2732; 1d p.i. *S*. Typhimurium- Jaccard: *F*_1,39_ = 7.614, *P* = 0.006399; Bray-Curtis: *F*_1,39_ = 9.1036, *P =* 0.003399; UW-UF: *F*_1,39_ = 2.3871, *P* = 0.1334; W-UF: *F*_1,39_ = 4.7569, *P* = 0.03379). The beta diversity within genotypes was approximated by the distance of each sample to the centroid of its respective cluster (*B6* ^+/-^ or *B6* ^-/-^).
(TIF)

S11 Fig Co-occurrence network after streptomycin and *S*. Typhimurium infection. (**A**) Distribution of pairwise genera correlations after *Salmonella* infection, with a higher number of weak negative interactions, but higher positive interaction strength (positive/negative interactions = 0.4381; *W* = 74056, *P* < 2.20 × 10^−16^; Wilcoxon test). (**B**) Genera co-occrurence network with highlighted indicators for *B6* genotypes. The network also visualizes the central and strong influence of *Salmonella* on other community members (square - *B6* ^+/-^ indicator, rectangle—*B6* ^-/-^ indicator, circle—no indicator/neutral; see S6 Table).
(TIF)

S1 Table Statistical analyses of CFU counts, cecum weights, inflammation markers, and gene expression. (DOC)

S2 Table Analyses of community resistance/turnover as community distance between pre- and post-infection time points in SPF raised mice. (DOC)

S3 Table Results of distance based redundancy analysis on different beta diversity metrics before and after *S*. Typhimurium infection. (DOC)

S4 Table Indicator species analysis based on consensus genera for *B4Galnt2* expression patterns in SPF mice (*B6*, *RIII*), before and after *S*. *Typhimurium* treatment. (DOC)

S5 Table Indicator species analysis based on species level OTUs for *B4Galnt2* genotypes in SPF mice (*B6*, *RIII*), before and after *S*. *Typhimurium* treatment. (DOC)

S6 Table Correlation of indicator genera to the rest of the pre-infection microbial community based on Spearman rank correlations (see S10 Fig). (DOC)

S7 Table Correlation of consensus genera- and species level OTU abundance before and after *S*. Typhimurium infection to the final histological inflammation score. (DOC)
