# Supplementary figures and images for: Expression of the Blood-Group-Related Gene B4galnt2 Alters Susceptibility to Salmonella Infection
Source: PLoS Pathog. 2015 Jul 2;11(7):e1005008. doi: 10.1371/journal.ppat.1005008 (PMC4489644; doi:10.1371/journal.ppat.1005008)

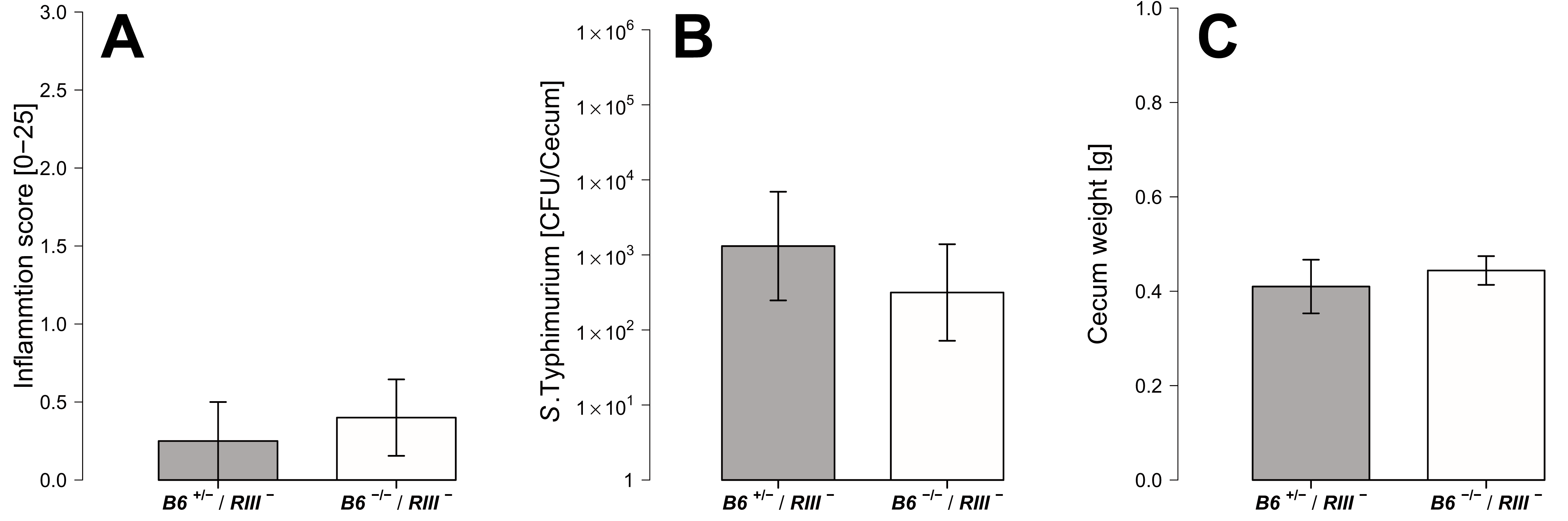

Supplement: S1 Fig — (A) We find no difference between mice differing in B4galnt2 expression in histological inflammation (Z = 0.447, P = 1.000), Salmonella load (B; Z = -0.747, P = 0.5658) and (C) cecum weight (Z = 0.490, P = 0.7311) (Wilcoxon test via Monte-Carlo resampling; # P < 0.100, * P < 0.050, ** P < 0.010, *** P < 0.001). (TIF) [file ppat.1005008.s001.tif]

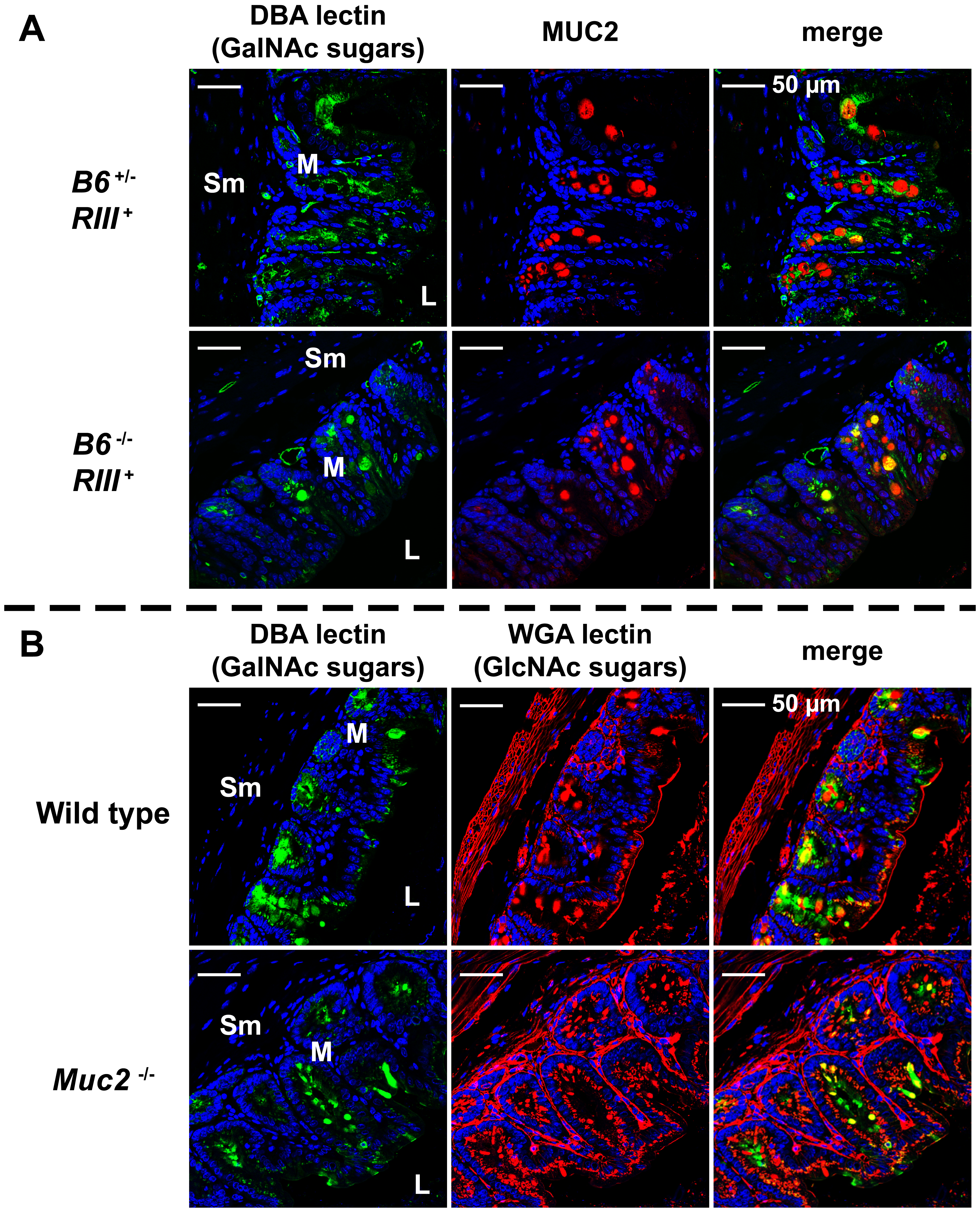

Supplement: S2 Fig — (A) Mucin-2 (MUC2) and B4galnt2 glycan residues (GalNAc) were stained with fluorescein labeled DBA in formalin fixed cecal tissue sections (Sm-submucosa, M-mucosa, L-lumen). (B) B4galnt2 glycan residues (GalNAc) were stained with fluorescein labeled DBA in formalin fixed cecal tissue sections before and 1 days p.i. with S. Typhimurium. GlcNAc residues were stained with Alexa633 labeled Wheat Germ Agglutinin (WGA). (TIF) [file ppat.1005008.s002.tif]

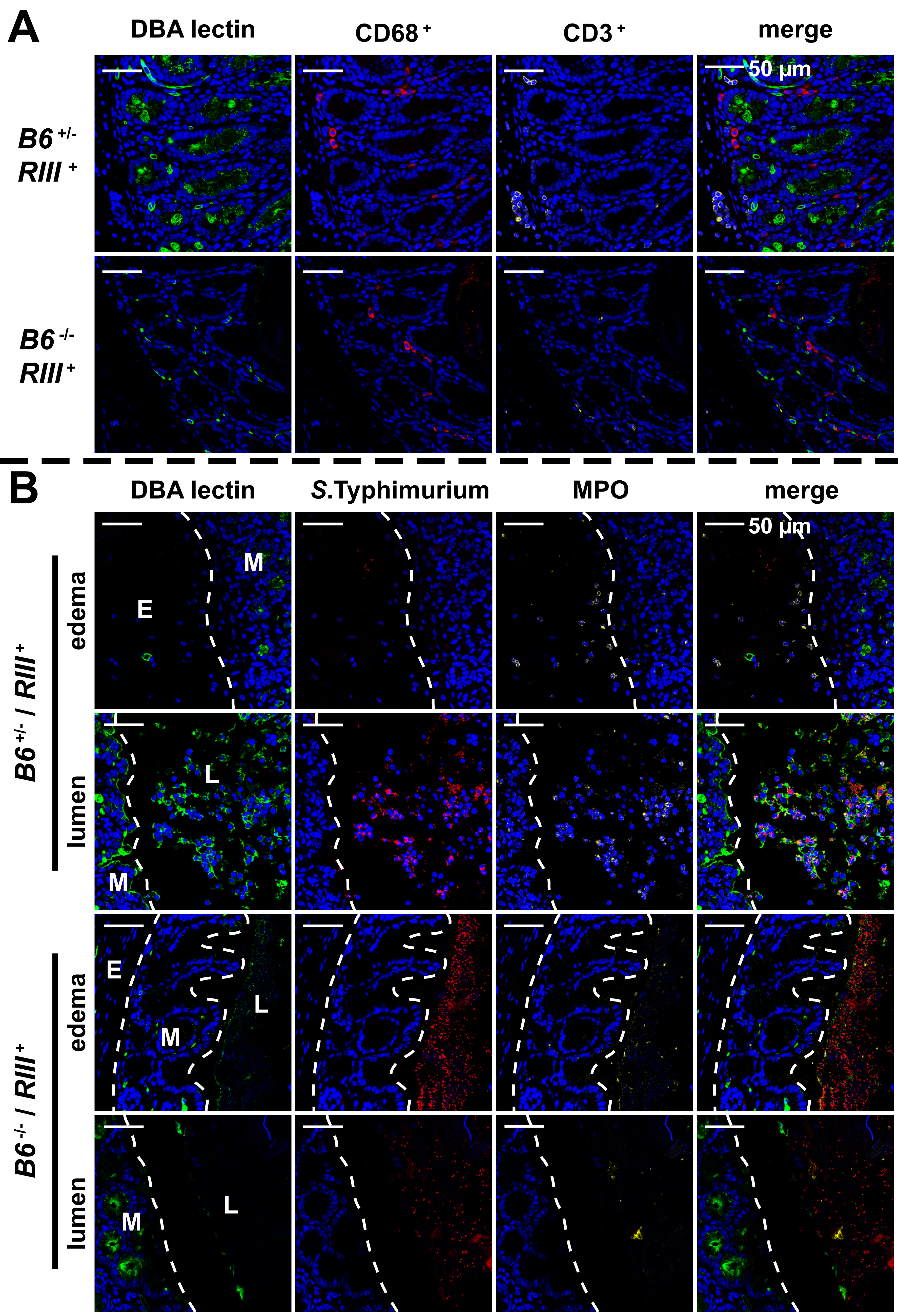

Supplement: S3 Fig — (A) Immunofluorescence staining and enumeration of positive cells per vision field showed that B6 +/- mice have higher numbers of CD68 + and CD3 + cells in the cecal mucosa 1d p.i. (N = 5–7; E-edema, M-mucosa, L-lumen). Nuclei were counterstained with DAPI and B4galnt2 glycans by using fluorescein labeled DBA. (B) Myeloperoxidase (MPO) positive cells and S. Typhimurium were determined by immunofluorescence staining in formalin fixed cecal sections (5 μm). (TIF) [file ppat.1005008.s003.tif]

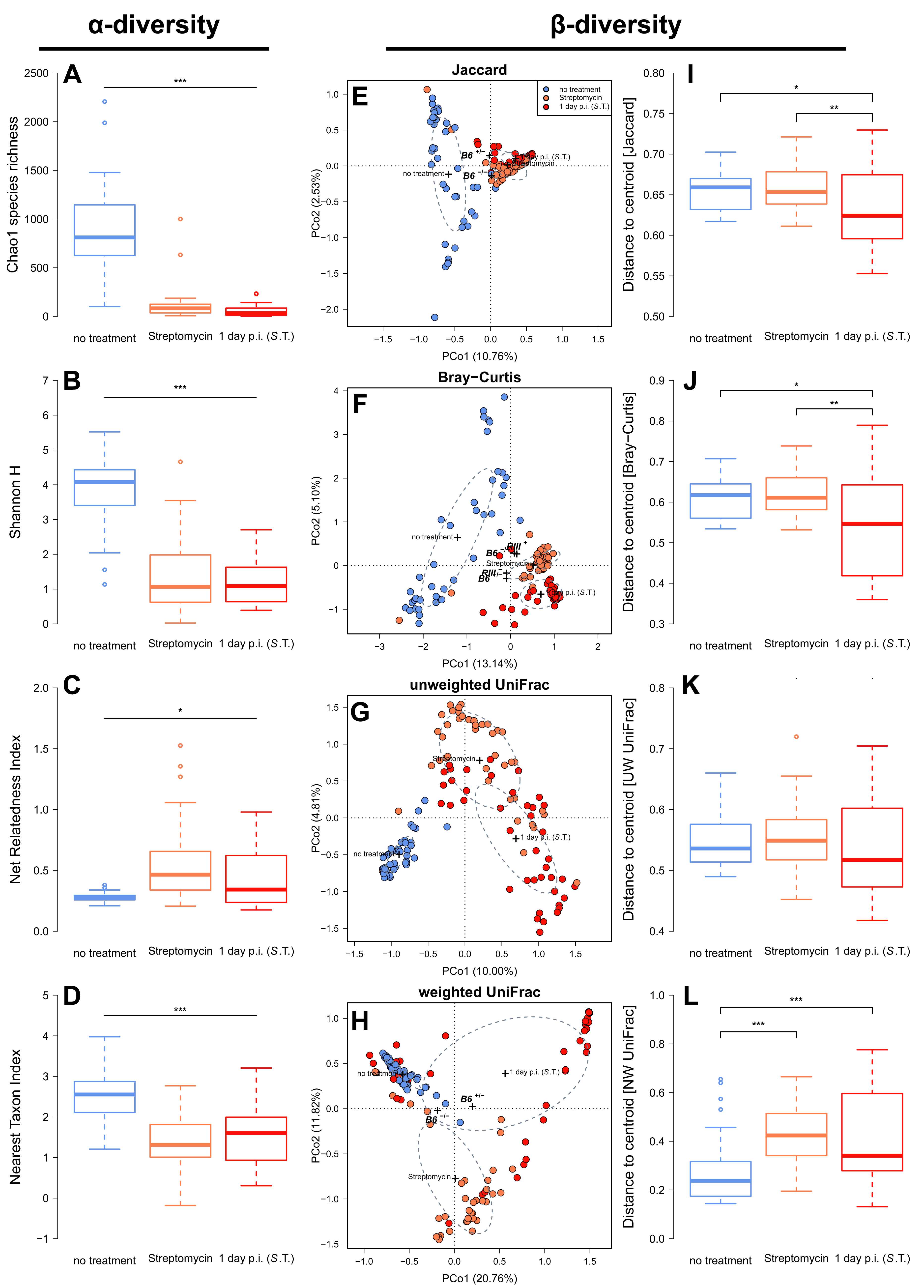

Supplement: S4 Fig — Microbial diversity was estimated from 97% species level OTUs and focused on species richness (A; Chao1: χ 2 = 78.940, P<2.2 × 10^−16^; Kruskal-Wallis test), species distribution (B; Shannon H: χ 2 = 65.997, P = 4.666 × 10^−15^; Kruskal-Wallis test), and distant and close phylogenetic relatedness (C; NRI: χ 2 = 6.4166, P = 0.04043; D; NTI: χ 2 = 50.4593, P = 1.104 × 10^−11^; Kruskal-Wallis test). Community changes among treatments were measured by the Jaccard distance (E; adonis: F 2,120 = 9.577, R 2 = 0.13765, P<0.0001), Bray-Curtis (F; adonis: F 2,120 = 12.055, R 2 = 0.1673, P<0.0001), UW-UF (G; adonis: F 2,120 = 13.932, R 2 = 0.18845, P<0.0001), and W-UF (H; adonis: F 2,120 = 20.615, R 2 = 0.25572, P<0.0001). Within treatment community variability (I-L) was also strongly influenced by the treatment regime (J- F 2,120 = 5.5668, P = 0.0054; BC- F 2,120 = 9.1942, P = 0.0004; W-UF: F 2,120 = 11.832, P<0.0001; UW-UF: F 2,120 = 1.7496, P = 0.1804) (TIF) [file ppat.1005008.s004.tif]

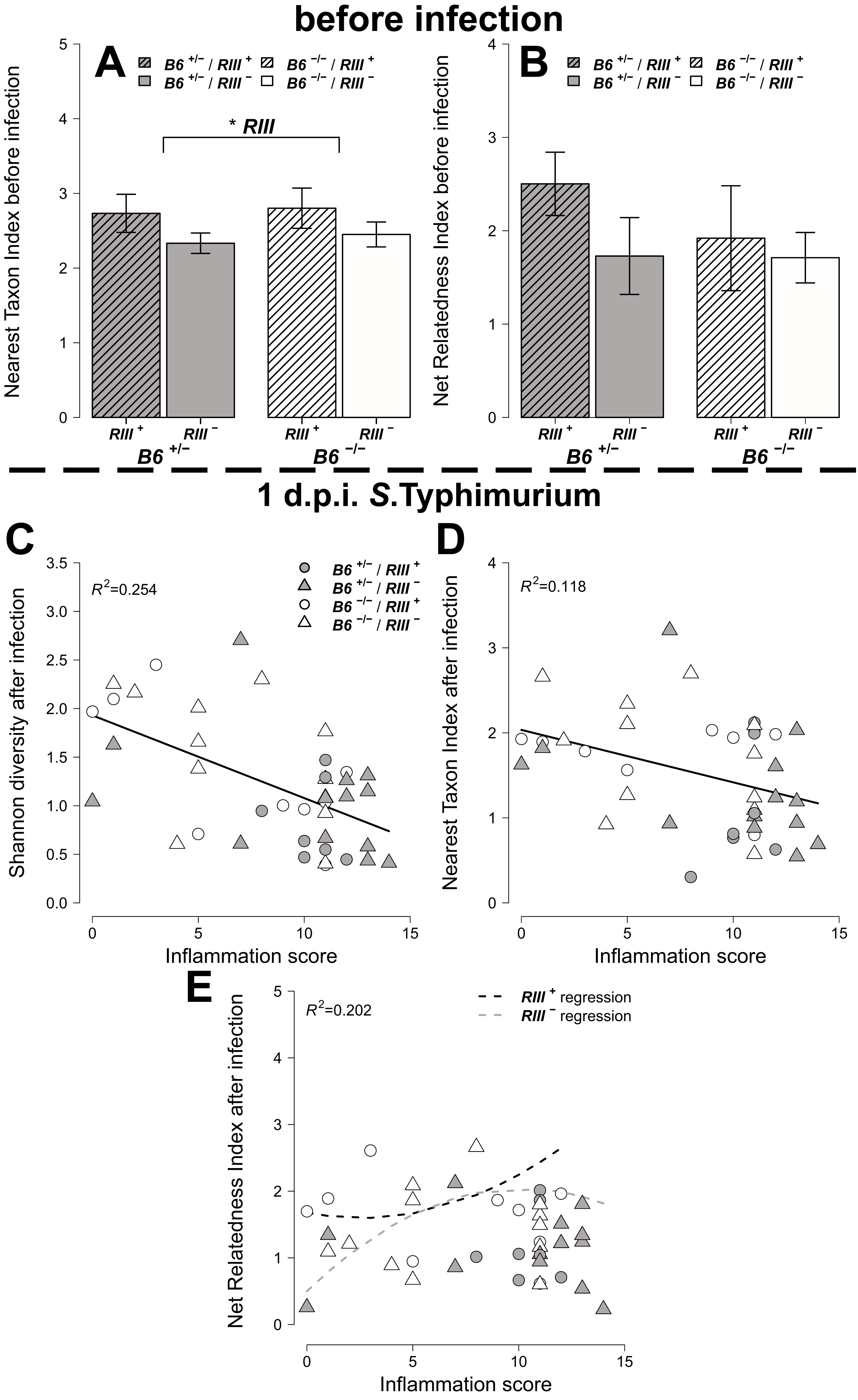

Supplement: S5 Fig — Microbial diversity was estimated from 97% species level OTUs and focused on species distribution (Shannon H: C), and close and distant phylogenetic relatedness (NTI: A, D; NRI: B, E), in the untreated state (A, B) and 1 day post infection with S. Typhimurium (C-E; Table 1 for the respective statistics). (TIF) [file ppat.1005008.s005.tif]

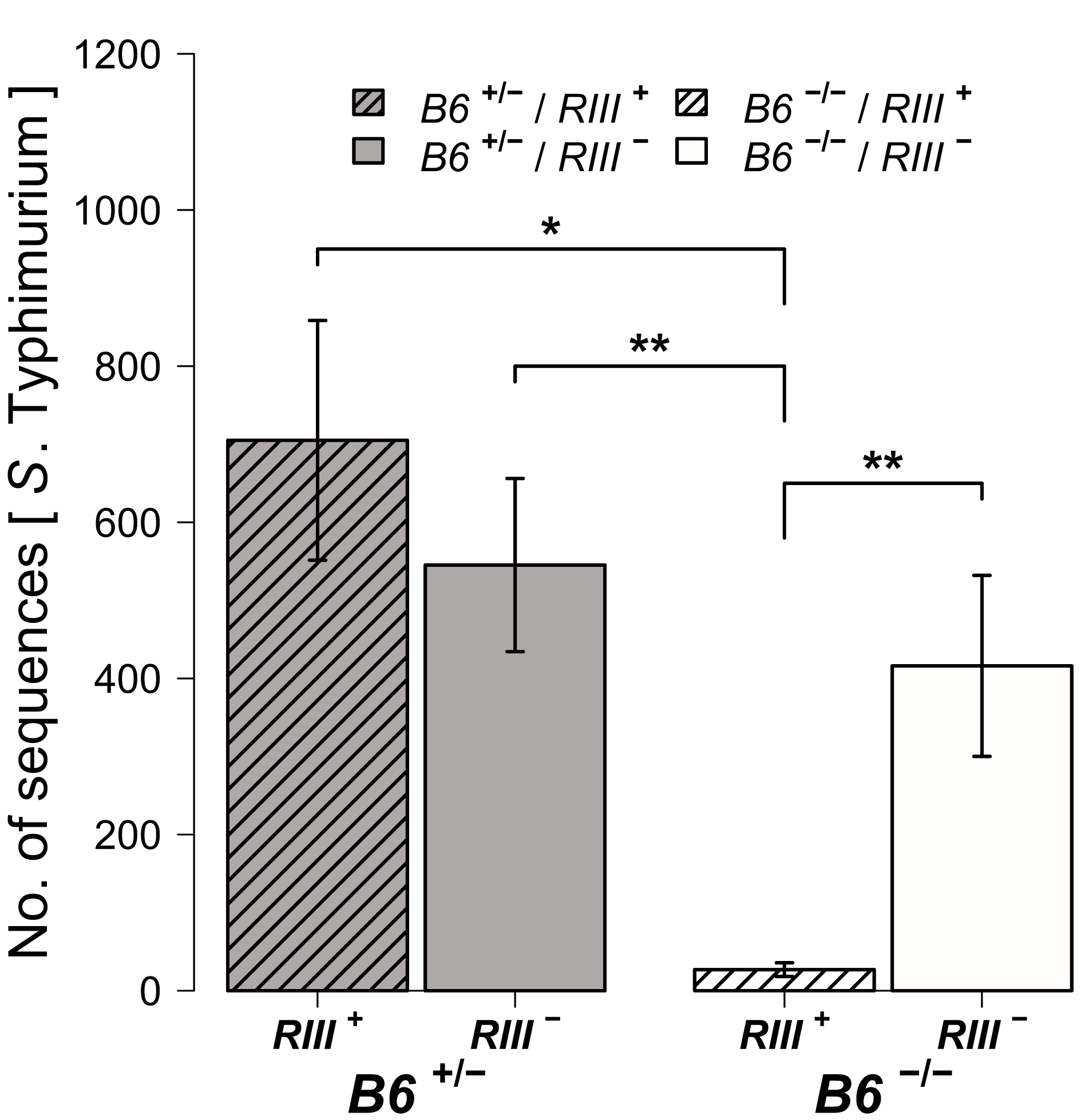

Supplement: S6 Fig — Salmonella abundance significantly differed between B6 and RIII genotypes (B6: F 1,20 = 5.32081, P = 0.0319; RIII: F 1,20 = 6.91949, P = 0.0160; B6/RIII: F 1,20 = 2.74565, P = 0.1131, R 2 LR = 0.28114; LMM) with the lowest abundance in RIII +/B6 -/- animals (Tukey pairwise comparisons: RIII +/B6 -/— RIII -/B6 -/-: Z = -3.102, P = 0.00979; RIII +/B6 -/— RIII -/B6 +/-: Z = -3.430, P = 0.00341; RIII +/B6 +/— RIII +/B6 -/-: Z = 2.582, P = 0.04698) (TIF) [file ppat.1005008.s006.tif]

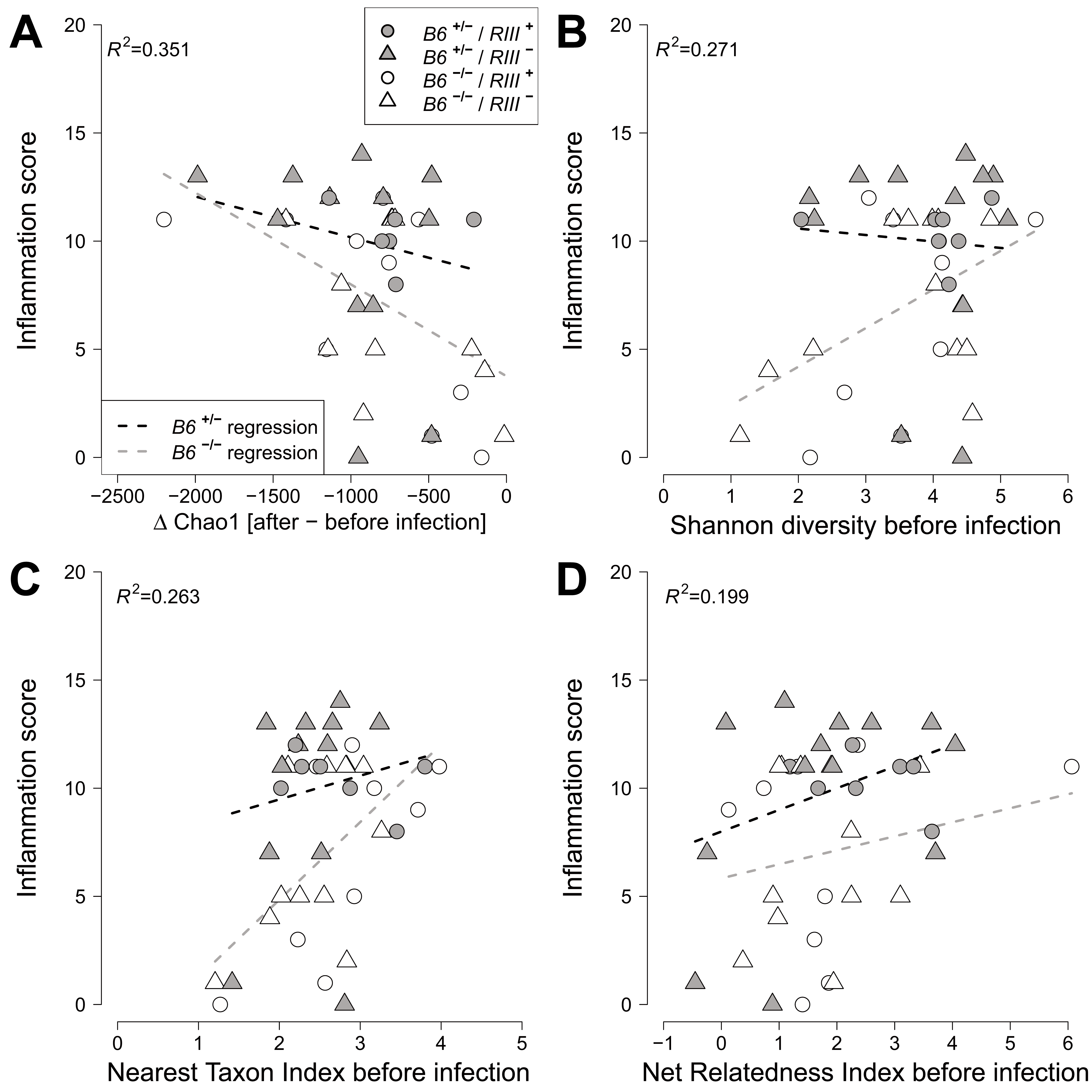

Supplement: S7 Fig — The severity of histological inflammation was significantly predictable by the change of species richness inflicted by S. Typhimurium infection and streptomycin treatment (A, ΔChao1), by the eveness of species distribution before infection (B, Shannon H), and clusteredness of closely related phylogenetic groups before infection (C, NTI). Phylogenetic clustering of distantly related species before infecation shows no significant association to the resulting inflammation (D, NRI, see Table 2). (TIF) [file ppat.1005008.s007.tif]

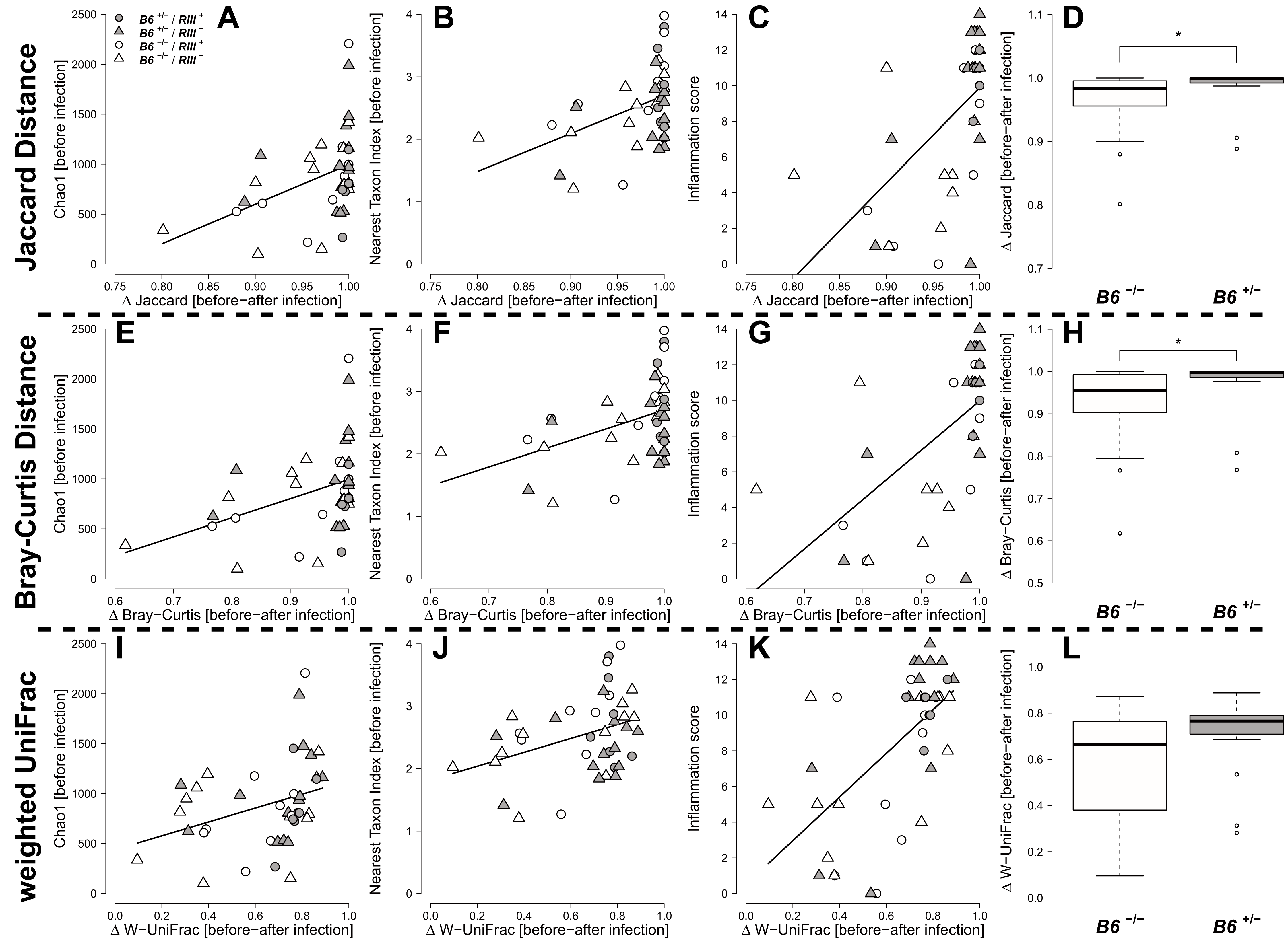

Supplement: S8 Fig — The community distances between animals before and after treatment were used as a measure of community disturbance, considering (A-D) species composition/Jaccard, (E-H) species abundance/Bray-Curtis, and (I-L) phylogenetic composition/weighted UniFrac. This disturbance signifies an increased species turnover (higher distance) in animals with a diverse microbial community measured in different ways, considering species number, distribution and phylogenetic relatedness (e.g. Chao1 (A, E, I), Nearest Taxon Index (B, F, J); see also S2 Table). Community turnover also correlates strongly with severity of inflammation, and increased Salmonella load (see S2 Table). Furthermore animals lacking epithelial B4galnt2 expression have on average less disturbance/higher resilience than mice with gut epithelial expression (D: Δ Jaccard: Z = -2.2731, P = 0.02311; H: Δ Bray-Curtis: Z = -2.2998, P = 0.0205; L: Δ W-UniFrac: Z = -1.6171, P = 0.1090; Wilcoxon test via Monte-Carlo resampling; see also Fig 7). (TIF) [file ppat.1005008.s008.tif]

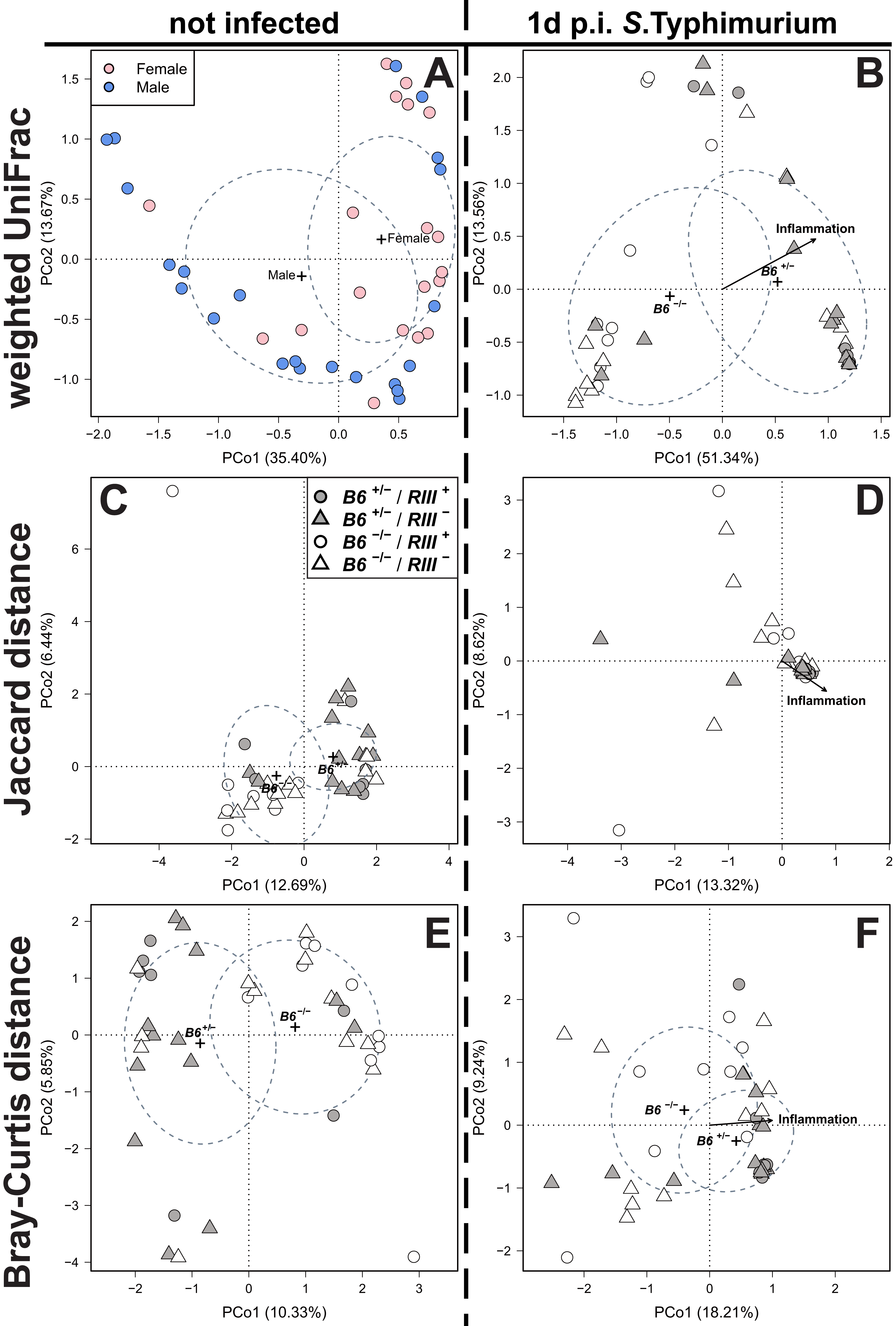

Supplement: S9 Fig — PCoAs of phylogenetically informed (A, B) and species based (C-F) metrics of beta diversity, that show clustering of microbial communities by epithelial B4galnt2 expression (C: R 2 = 0.1478, P = 0.0011; E: R 2 = 0.1373, P = 0.0020) and sex (A: R 2 = 0.0884, P = 0.0260) before any treatment. After S. Typhimurium infection the community structures show strong and consistent correlation to histological inflammation (B: R 2 = 0.5054, P<0.0001; D: R 2 = 0.3167, P = 0.0006; F: R 2 = 0.4935, P = 0.0002) and significant discrimination among epithelial and endothelial B4galnt2 expression patterns (B: B6-R 2 = 0.1272, P = 0.005199; F: B6-R 2 = 0.0951, P = 0.01430). (TIF) [file ppat.1005008.s009.tif]

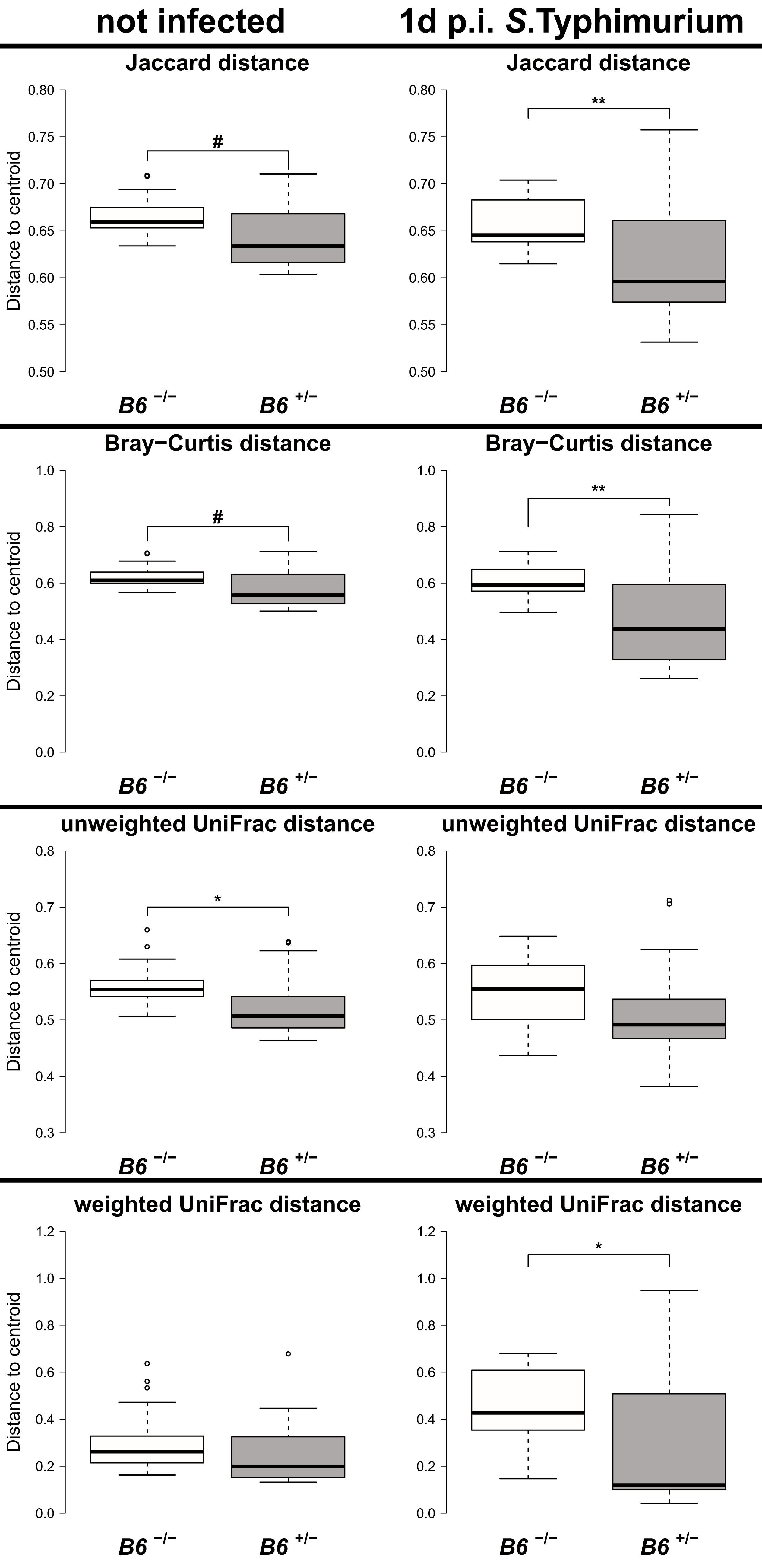

Supplement: S10 Fig — Comparison of bacterial community distances (beta diversity) between animals with and without epithelial B4Galnt2 expression, before and after S. Typhimurium infection (not infected- Jaccard: F 1,39 = 4.1584, P = 0.04779; Bray-Curtis: F 1,39 = 3.961, P = 0.05379, UW-UF: F 1,39 = 5.414, P = 0.0246; W-UF: F 1,39 = 1.235, P = 0.2732; 1d p.i. S. Typhimurium- Jaccard: F 1,39 = 7.614, P = 0.006399; Bray-Curtis: F 1,39 = 9.1036, P = 0.003399; UW-UF: F 1,39 = 2.3871, P = 0.1334; W-UF: F 1,39 = 4.7569, P = 0.03379). The beta diversity within genotypes was approximated by the distance of each sample to the centroid of its respective cluster (B6 +/- or B6 -/-). (TIF) [file ppat.1005008.s010.tif]

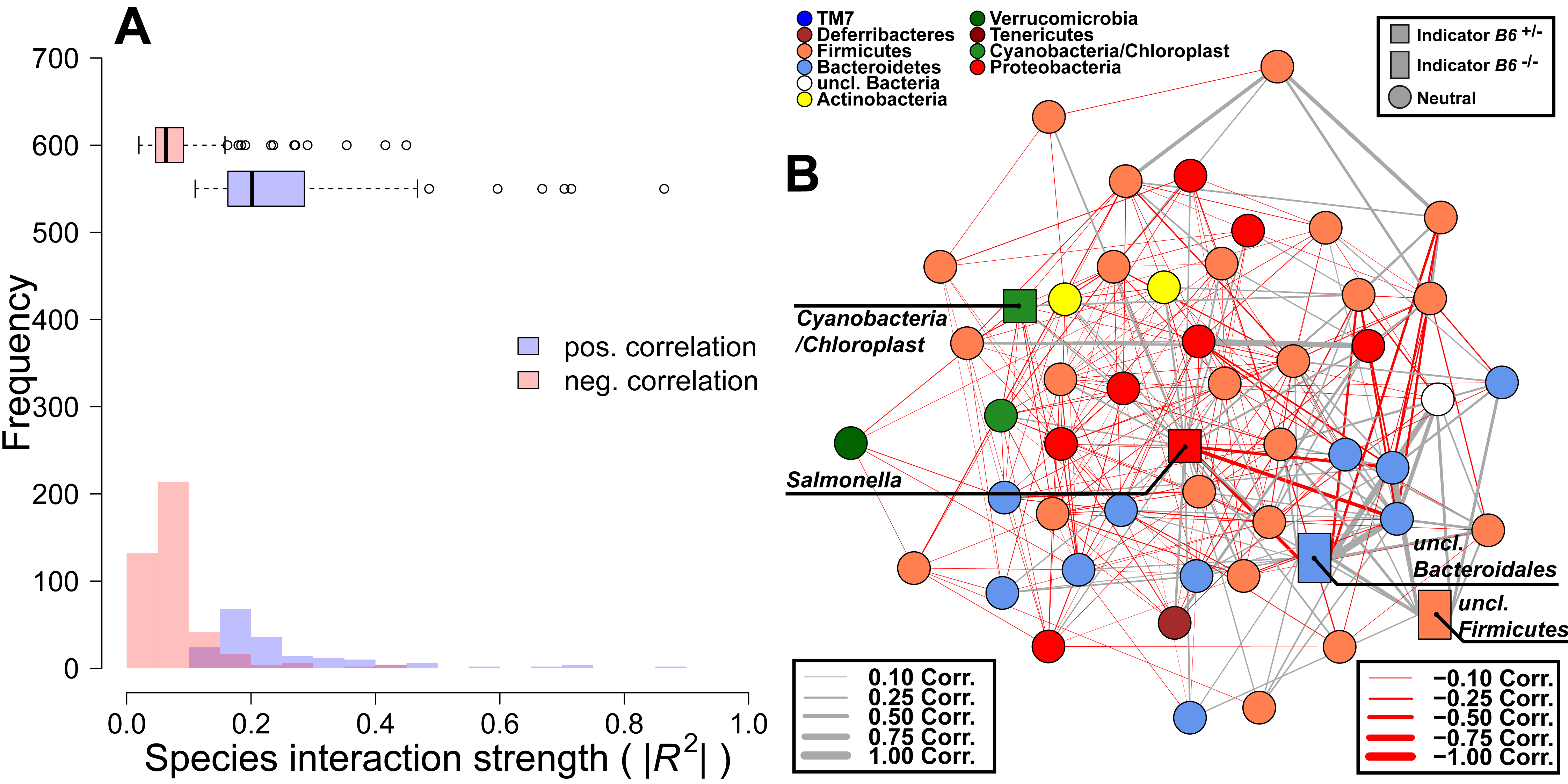

Supplement: S11 Fig — (A) Distribution of pairwise genera correlations after Salmonella infection, with a higher number of weak negative interactions, but higher positive interaction strength (positive/negative interactions = 0.4381; W = 74056, P < 2.20 × 10^−16^; Wilcoxon test). (B) Genera co-occrurence network with highlighted indicators for B6 genotypes. The network also visualizes the central and strong influence of Salmonella on other community members (square - B6 +/- indicator, rectangle—B6 -/- indicator, circle—no indicator/neutral; see S6 Table). (TIF) [file ppat.1005008.s011.tif]
