# Supplementary material for: Expression of the Blood-Group-Related Gene B4galnt2 Alters Susceptibility to Salmonella Infection
Source: PLoS Pathog. 2015 Jul 2;11(7):e1005008. doi: 10.1371/journal.ppat.1005008 (PMC4489644; doi:10.1371/journal.ppat.1005008)
Supplement: S1 Table — (DOC) [file ppat.1005008.s012.doc]

|  | not infected | | | | 1 day p.i. | | | | 14 days p.i. | | | |
| --- | --- | --- | --- | --- | --- | --- | --- | --- | --- | --- | --- | --- |
| Measurement | Factors | *Df* | *F*-Value | *P*-Value | Factors | *Df* | *F*-Value | *P*-Value | Factors | *Df* | *F*-Value | *P*-Value |
| Lipocalin-2 (Lcn-2)** | *1* | 16 | NA | NA | *B6* | 1,28 | 17.494 | 0.0003 | - | - | - | - |
|  |  |  |  |  | *RIII* | 1,28 | 7.271 | 0.0117 | - | - | - | - |
| Colony Forming Units* | - | - | - | - | *RIII* | 1,49 | 10.537 | 0.0021 | *B6* | 1,23 | 0.0098 | 0.92207 |
|  | - | - | - | - |  |  |  |  | *RIII* | 1,23 | 1.1159 | 0.30176 |
|  | - | - | - | - |  |  |  |  | *B6*: *RIII* | 1,23 | 7.3680 | 0.01237 |
| CD3 cells* | - | - | - | - | *B6* | 1,22 | 20.170 | 0.0002 | - | - | - | - |
| CD68 cells | - | - | - | - | *B6* | 1,22 | 19.060 | 0.0003 | - | - | - | - |
| MPO (RFU signal) | - | - | - | - | *B6* | 1,26 | 20.300 | 0.0001 | - | - | - | - |

* log(X); ** X^1/4^; *** X^2^ data transformations; NA - no data available
