# Supplementary material for: Expression of the Blood-Group-Related Gene B4galnt2 Alters Susceptibility to Salmonella Infection
Source: PLoS Pathog. 2015 Jul 2;11(7):e1005008. doi: 10.1371/journal.ppat.1005008 (PMC4489644; doi:10.1371/journal.ppat.1005008)
Supplement: S2 Table — (DOC) [file ppat.1005008.s013.doc]

| Distance | Factor | *ρ* | *P*-Value | Comparison | *Z* | *P*-Value |
| --- | --- | --- | --- | --- | --- | --- |
| Bray-Curtis | Inflammation | 0.6131 | **0.000020** | *B6* | -2.2998 | **0.0205** |
| (no-1 d.p.i.) | *Salmonella* | 0.6393 | **0.000007** | *RIII* | -1.0139 | 0.3204 |
|  | Chao1 (no treatment) | 0.5062 | **0.000733** |  |  |  |
|  | Shannon H (no treatment) | 0.4332 | **0.004670** |  |  |  |
|  | NRI (no treatment) | 0.0922 | 0.566500 |  |  |  |
|  | NTI (no treatment) | 0.3631 | **0.019610** |  |  |  |
|  | ΔChao1 (1 d.p.i.-no treat.) | -0.5911 | **0.000047** |  |  |  |
| Jaccard | Inflammation | 0.6092 | **0.000024** | *B6* | -2.2731 | **0.0231** |
| (no-1 d.p.i.) | *Salmonella* | 0.6236 | **0.000013** | *RIII* | -1.0139 | 0.3227 |
|  | Chao1 (no treatment) | 0.5266 | **0.000405** |  |  |  |
|  | Shannon H (no treatment) | 0.4484 | **0.003283** |  |  |  |
|  | NRI (no treatment) | 0.0768 | 0.633100 |  |  |  |
|  | NTI (no treatment) | 0.3785 | **0.014680** |  |  |  |
|  | ΔChao1 (1 d.p.i.-no treat.) | -0.6098 | **0.000023** |  |  |  |
| unweighted | Inflammation | 0.5894 | **0.000050** | *B6* | -2.3213 | **0.0198** |
| UniFrac | *Salmonella* | 0.6024 | **0.000031** | *RIII* | -0.3742 | 0.7216 |
| (no-1 d.p.i.) | Chao1 (no treatment) | 0.6040 | **0.000042** |  |  |  |
|  | Shannon H (no treatment) | 0.5057 | **0.000869** |  |  |  |
|  | NRI (no treatment) | 0.1030 | 0.520500 |  |  |  |
|  | NTI (no treatment) | 0.2920 | 0.064310 |  |  |  |
|  | ΔChao1 (1 d.p.i.-no treat.) | -0.6897 | **0.000001** |  |  |  |
| weighted | Inflammation | 0.5429 | **0.000245** | *B6* | -1.6171 | 0.1090 |
| UniFrac | *Salmonella* | 0.7412 | **0.000000** | *RIII* | -0.1871 | 0.8649 |
| (no-1 d.p.i.) | Chao1 (no treatment) | 0.4469 | **0.003701** |  |  |  |
|  | Shannon H (no treatment) | 0.4132 | **0.007664** |  |  |  |
|  | NRI (no treatment) | 0.2145 | 0.177600 |  |  |  |
|  | NTI (no treatment) | 0.3334 | **0.033660** |  |  |  |
|  | ΔChao1 (1 d.p.i.-no treat.) | -0.5148 | **0.000680** |  |  |  |
