# Supplementary material for: Expression of the Blood-Group-Related Gene B4galnt2 Alters Susceptibility to Salmonella Infection
Source: PLoS Pathog. 2015 Jul 2;11(7):e1005008. doi: 10.1371/journal.ppat.1005008 (PMC4489644; doi:10.1371/journal.ppat.1005008)
Supplement: S3 Table — (DOC) [file ppat.1005008.s014.doc]

| Time point | Distance | Factor | *DF* | *F*-Value | *P*-Value | *R*^2^ | adj. *R*^2^ |
| --- | --- | --- | --- | --- | --- | --- | --- |
| before | UW-UniFrac | *B6* | 1,39 | 1.6603 | 0.0008 | 0.0408 | 0.0162 |
| treatment | NW-UniFrac | *B6* | 1,39 | 1.3449 | 0.1336 | 0.0333 | 0.0085 |
|  | Jaccard | *B6* | 1,39 | 1.6369 | 0.0006 | 0.0403 | 0.0157 |
|  | Bray-Curtis | *B6* | 1,39 | 2.2698 | 0.0010 | 0.0550 | 0.0308 |
|  | RDA | *B6* | 1,39 | 2.3381 | 0.0028 | 0.0566 | 0.0324 |
| 1 d.p.i. | UW-UniFrac | *B6* | 1,39 | 1.4054 | 0.0402 | 0.0348 | 0.0100 |
|  | NW-UniFrac | *B6* | 1,39 | 4.5133 | 0.0028 | 0.1078 | 0.0849 |
|  | Jaccard | *B6* | 1,39 | 1.5109 | 0.0114 | 0.0373 | 0.0126 |
|  | Bray-Curtis | *B6* | 1,39 | 2.7568 | 0.0034 | 0.0664 | 0.0424 |
|  | RDA | *B6* | 1,39 | 3.1621 | 0.0058 | 0.0750 | 0.0513 |
|  | UW-UniFrac | *RIII* | 1,37 | 1.2393 | 0.0942 | 0.1351 | 0.0650 |
|  |  | *B6* | 1,37 | 1.5350 | 0.0180 |  |  |
|  |  | Inflammation | 1,37 | 3.0064 | 0.0002 |  |  |
|  | NW-UniFrac | *RIII* | 1,37 | 1.2393 | 0.0996 | 0.1351 | 0.0650 |
|  |  | *B6* | 1,37 | 1.5350 | 0.0186 |  |  |
|  |  | Inflammation | 1,37 | 3.0064 | 0.0002 |  |  |
|  | Jaccard | *RIII* | 1,37 | 1.0892 | 0.2424 | 0.1197 | 0.0483 |
|  |  | *B6* | 1,37 | 1.6055 | 0.0094 |  |  |
|  |  | Inflammation | 1,37 | 2.3346 | 0.0002 |  |  |
|  | Bray-Curtis | *RIII* | 1,37 | 1.2220 | 0.1866 | 0.1901 | 0.1244 |
|  |  | *B6* | 1,37 | 2.9999 | 0.0020 |  |  |
|  |  | Inflammation | 1,37 | 4.4022 | 0.0002 |  |  |
|  | RDA | *RIII* | 1,37 | 1.5673 | 0.1046 | 0.2140 | 0.1503 |
|  |  | *B6* | 1,37 | 3.3826 | 0.0050 |  |  |
|  |  | Inflammation | 1,37 | 5.1250 | 0.0002 |  |  |
