# Supplementary material for: Expression of the Blood-Group-Related Gene B4galnt2 Alters Susceptibility to Salmonella Infection
Source: PLoS Pathog. 2015 Jul 2;11(7):e1005008. doi: 10.1371/journal.ppat.1005008 (PMC4489644; doi:10.1371/journal.ppat.1005008)
Supplement: S4 Table — (DOC) [file ppat.1005008.s015.doc]

| Time point | Classification (RDP 9, modified by P.Schloss) | Factor | *r.g.* | *P*-Value | *P*-Value (FDR) |
| --- | --- | --- | --- | --- | --- |
| before | *Barnesiella* | *B6*^-/-^ | 0.31314 | 0.02480 | 0.25624 |
| treatment | *unclassified Porphyromonadaceae* | *B6*^-/-^ | 0.41552 | 0.00390 | 0.06044 |
|  | *Turicibacter* | *B6*^-/-^ | 0.42849 | 0.00030 | 0.01860 |
|  | *Bacteroides* | *B6*^+/-^ | 0.42503 | 0.00280 | 0.05786 |
|  | *Parasutterella* | *B6*^+/-^ | 0.33333 | 0.04970 | 0.44016 |
|  | *Prevotella* | *B6*^+/-^ | 0.41335 | 0.00880 | 0.10911 |
|  | *unclassified Prevotellaceae* | *B6*^+/-^ | 0.49092 | 0.00160 | 0.04960 |
| 1 d.p.i. | *unclassified Bacteroidales* | *B6*^-/-^ | 0.33913 | 0.03040 | 0.37631 |
|  | *unclassified Firmicutes* | *B6*^-/-^ | 0.31522 | 0.03580 | 0.37631 |
|  | *Salmonella* | *B6*^+/-^ | 0.42981 | 0.00580 | 0.22618 |
|  | *Streptophyta* | *B6*^+/-^ | 0.33049 | 0.03860 | 0.37631 |
| before | *unclassified Erysipelotrichaceae* | *RIII*^+^ | 0.43033 | 0.00640 | 0.19838 |
| treatment | *Marvinbryantia* | *RIII*^+^ | 0.34085 | 0.02800 | 0.57861 |
|  | *Turicibacter* | *RIII*^+^ | 0.40214 | 0.00110 | 0.06819 |
| 1 d.p.i. | *unclassified Lachnospiraceae* | *RIII*^+^ | 0.19198 | 0.01900 | 0.66215 |
|  | *Turicibacter* | *RIII*^+^ | 0.20854 | 0.04350 | 0.66215 |
