# Supplementary material for: Expression of the Blood-Group-Related Gene B4galnt2 Alters Susceptibility to Salmonella Infection
Source: PLoS Pathog. 2015 Jul 2;11(7):e1005008. doi: 10.1371/journal.ppat.1005008 (PMC4489644; doi:10.1371/journal.ppat.1005008)
Supplement: S5 Table — (DOC) [file ppat.1005008.s016.doc]

| Time point | Classification (RDP 9, modified by P.Schloss) | Factor | *r.g.* | *P*-Value | *P*-Value (FDR) |
| --- | --- | --- | --- | --- | --- |
| before | *0010-Firmicutes(100);Erysipelotrichia(100);Erysipelotrichales(100);Erysipelotrichaceae(100);Turicibacter(100);* | *B6* ^-/-^ | 0.4151 | 0.0005 | 0.2095 |
| treatment | *0013-Bacteroidetes(100);Bacteroidia(94);Bacteroidales(94);Porphyromonadaceae(87);Barnesiella(66);* | *B6* ^-/-^ | 0.4734 | 0.0005 | 0.2095 |
|  | *0050-Firmicutes(100);Clostridia(100);Clostridiales(100);Lachnospiraceae(100);Robinsoniella(96);* | *B6* ^-/-^ | 0.4043 | 0.0034 | 0.4383 |
|  | *0053-Bacteroidetes(100);Bacteroidia(100);Bacteroidales(100);Porphyromonadaceae(100);Butyricimonas(66);* | *B6* ^-/-^ | 0.4427 | 0.0013 | 0.2607 |
|  | *0101-Bacteroidetes(100);Bacteroidia(97);Bacteroidales(97);Porphyromonadaceae(97);Paludibacter(96);* | *B6* ^-/-^ | 0.3598 | 0.0072 | 0.5538 |
|  | *0125-Firmicutes(100);Clostridia(100);Clostridiales(100);Lachnospiraceae(100);Robinsoniella(96);* | *B6* ^-/-^ | 0.2386 | 0.0449 | 0.8453 |
|  | *0157-Firmicutes(100);Clostridia(100);Clostridiales(100);Ruminococcaceae(98);Hydrogenoanaerobacterium(91);* | *B6* ^-/-^ | 0.4036 | 0.0082 | 0.5608 |
|  | *0189-Firmicutes(100);Clostridia(100);Clostridiales(100);Lachnospiraceae(100);Lachnobacterium(98);* | *B6* ^-/-^ | 0.2080 | 0.0073 | 0.5538 |
|  | *0195-Bacteroidetes(100);Bacteroidia(89);Bacteroidales(89);Porphyromonadaceae(89);Paludibacter(53);* | *B6* ^-/-^ | 0.3612 | 0.0146 | 0.7134 |
|  | *0196-Bacteroidetes(100);Flavobacteria(53);Flavobacteriales(53);Flavobacteriaceae(53);Pseudozobellia(53);* | *B6* ^-/-^ | 0.3631 | 0.0061 | 0.5538 |
|  | *0215-Bacteroidetes(100);Bacteroidia(100);Bacteroidales(100);Porphyromonadaceae(100);Tannerella(59);* | *B6* ^-/-^ | 0.4028 | 0.0029 | 0.4050 |
|  | *0216-Bacteroidetes(100);Bacteroidia(97);Bacteroidales(97);Marinilabiaceae(83);Anaerophaga(83);* | *B6* ^-/-^ | 0.3159 | 0.0450 | 0.8453 |
|  | *0252-Bacteroidetes(100);Bacteroidia(92);Bacteroidales(92);Marinilabiaceae(63);Anaerophaga(63);* | *B6* ^-/-^ | 0.3349 | 0.0067 | 0.5538 |
|  | *0276-Firmicutes(100);Clostridia(100);Clostridiales(100);Lachnospiraceae(100);Lachnospiracea incertae sedis(96);* | *B6* ^-/-^ | 0.3711 | 0.0084 | 0.5608 |
|  | *0286-Firmicutes(100);Clostridia(100);Clostridiales(100);Peptococcaceae 1(96);Peptococcus(96);* | *B6* ^-/-^ | 0.2774 | 0.0464 | 0.8453 |
|  | *0287-Bacteroidetes(100);Sphingobacteria(55);Sphingobacteriales(55);Flammeovirgaceae(55);Limibacter(55);* | *B6* ^-/-^ | 0.3494 | 0.0076 | 0.5538 |
|  | *0293-Bacteroidetes(100);Bacteroidia(61);Bacteroidales(61);* | *B6* ^-/-^ | 0.3617 | 0.0204 | 0.8453 |
|  | *0317-Bacteroidetes(100);Bacteroidia(100);Bacteroidales(100);Porphyromonadaceae(100);Barnesiella(71);* | *B6* ^-/-^ | 0.4350 | 0.0020 | 0.3352 |
|  | *0356-Bacteroidetes(100);Bacteroidia(100);Bacteroidales(100);Porphyromonadaceae(100);Paludibacter(86);* | *B6* ^-/-^ | 0.2958 | 0.0487 | 0.8453 |
|  | *0358-Bacteroidetes(100);Bacteroidia(100);Bacteroidales(100);Marinilabiaceae(93);Anaerophaga(93);* | *B6* ^-/-^ | 0.3329 | 0.0460 | 0.8453 |
|  | *0361-Firmicutes(100);Clostridia(100);Clostridiales(100);Lachnospiraceae(100);Acetitomaculum(100);* | *B6* ^-/-^ | 0.3213 | 0.0488 | 0.8453 |
|  | *0370-Firmicutes(100);Clostridia(100);Clostridiales(100);Lachnospiraceae(100);Robinsoniella(93);* | *B6* ^-/-^ | 0.3392 | 0.0236 | 0.8453 |
|  | *0391-Firmicutes(100);Clostridia(100);Clostridiales(100);Ruminococcaceae(100);Flavonifractor(100);* | *B6* ^-/-^ | 0.3437 | 0.0324 | 0.8453 |
|  | *0420-Firmicutes(100);Erysipelotrichia(100);Erysipelotrichales(100);Erysipelotrichaceae(100);Turicibacter(100);* | *B6* ^-/-^ | 0.3859 | 0.0226 | 0.8453 |
|  | *0424-Firmicutes(100);Clostridia(100);Clostridiales(100);Peptococcaceae 1(100);Peptococcus(100);* | *B6* ^-/-^ | 0.3456 | 0.0175 | 0.7926 |
|  | *0512-Bacteroidetes(100);Bacteroidia(100);Bacteroidales(100);Porphyromonadaceae(89);* | *B6* ^-/-^ | 0.2872 | 0.0494 | 0.8453 |
|  | *0525-Bacteroidetes(100);Bacteroidia(100);Bacteroidales(100);Porphyromonadaceae(78);Paludibacter(78);* | *B6* ^-/-^ | 0.3361 | 0.0487 | 0.8453 |
|  | *0678-Bacteroidetes(100);Bacteroidia(100);Bacteroidales(100);Porphyromonadaceae(100);Barnesiella(100);* | *B6* ^-/-^ | 0.4082 | 0.0216 | 0.8453 |
|  | *0815-Firmicutes(100);Clostridia(100);Clostridiales(100);Ruminococcaceae(100);Butyricicoccus(100);* | *B6* ^-/-^ | 0.3676 | 0.0448 | 0.8453 |
|  | *0019-Bacteroidetes(100);Bacteroidia(97);Bacteroidales(97);Porphyromonadaceae(96);Tannerella(95);* | *B6*^+/-^ | 0.3155 | 0.0393 | 0.8453 |
|  | *0031-Bacteroidetes(100);Bacteroidia(100);Bacteroidales(100);Prevotellaceae(100);Prevotella(85);* | *B6*^+/-^ | 0.4956 | 0.0009 | 0.2514 |
|  | *0034-Bacteroidetes(100);Sphingobacteria(93);Sphingobacteriales(93);Flammeovirgaceae(93);Limibacter(93);* | *B6*^+/-^ | 0.3795 | 0.0145 | 0.7134 |
|  | *0074-Bacteroidetes(100);Bacteroidia(96);Bacteroidales(96);Porphyromonadaceae(96);Paludibacter(92);* | *B6*^+/-^ | 0.3838 | 0.0073 | 0.5538 |
|  | *0079-Bacteroidetes(100);Bacteroidia(70);Bacteroidales(70);Porphyromonadaceae(66);Paludibacter(66);* | *B6*^+/-^ | 0.3530 | 0.0247 | 0.8453 |
|  | *0082-Bacteroidetes(100);Bacteroidia(100);Bacteroidales(100);Bacteroidaceae(100);Bacteroides(100);* | *B6*^+/-^ | 0.3912 | 0.0076 | 0.5538 |
|  | *0090-Bacteroidetes(100);Bacteroidia(97);Bacteroidales(97);Porphyromonadaceae(92);Tannerella(92);* | *B6*^+/-^ | 0.3549 | 0.0149 | 0.7134 |
|  | *0092-Bacteroidetes(100);Bacteroidia(100);Bacteroidales(100);Rikenellaceae(100);Rikenella(100);* | ***B6*^+/-^** | **0.4292** | **0.0001** | **0.0838** |
|  | *0100-Firmicutes(100);Clostridia(100);Clostridiales(100);Lachnospiraceae(99);Robinsoniella(98);* | *B6*^+/-^ | 0.2492 | 0.0060 | 0.5538 |
|  | *0117-Bacteroidetes(100);Sphingobacteria(100);Sphingobacteriales(100);Flammeovirgaceae(100);Limibacter(100);* | *B6*^+/-^ | 0.3921 | 0.0069 | 0.5538 |
|  | *0120-Bacteroidetes(100);Bacteroidia(98);Bacteroidales(98);Porphyromonadaceae(98);Paludibacter(98);* | *B6*^+/-^ | 0.3845 | 0.0111 | 0.6001 |
|  | *0147-Bacteroidetes(100);Sphingobacteria(54);Sphingobacteriales(54);Flammeovirgaceae(54);Limibacter(54);* | *B6*^+/-^ | 0.3258 | 0.0229 | 0.8453 |
|  | *0149-Bacteroidetes(100);Bacteroidia(83);Bacteroidales(83);Porphyromonadaceae(83);Paludibacter(81);* | *B6*^+/-^ | 0.4141 | 0.0052 | 0.5538 |
|  | *0150-Bacteroidetes(100);Sphingobacteria(90);Sphingobacteriales(90);Flammeovirgaceae(90);Limibacter(90);* | *B6*^+/-^ | 0.3965 | 0.0014 | 0.2607 |
|  | *0153-Firmicutes(100);Clostridia(100);Clostridiales(100);Lachnospiraceae(100);Syntrophococcus(77);* | *B6*^+/-^ | 0.1881 | 0.0477 | 0.8453 |
|  | *0159-Bacteroidetes(100);Bacteroidia(80);Bacteroidales(80);Porphyromonadaceae(63);Paludibacter(56);* | *B6*^+/-^ | 0.3678 | 0.0183 | 0.8070 |
|  | *0161-Bacteroidetes(100);Bacteroidia(74);Bacteroidales(74);Marinilabiaceae(53);Anaerophaga(53);* | *B6*^+/-^ | 0.3256 | 0.0227 | 0.8453 |
|  | *0165-Bacteroidetes(98);Bacteroidia(52);Bacteroidales(52);* | ***B6*^+/-^** | **0.5930** | **0.0001** | **0.0838** |
|  | *0168-Bacteroidetes(100);Bacteroidia(66);Bacteroidales(66);Porphyromonadaceae(61);* | *B6*^+/-^ | 0.3764 | 0.0161 | 0.7495 |
|  | *0201-Bacteroidetes(100);Bacteroidia(76);Bacteroidales(76);Rikenellaceae(61);Rikenella(61);* | *B6*^+/-^ | 0.3816 | 0.0108 | 0.6001 |
|  | *0203-Bacteroidetes(100);Sphingobacteria(54);Sphingobacteriales(54);Flammeovirgaceae(54);Limibacter(54);* | *B6*^+/-^ | 0.4490 | 0.0026 | 0.3961 |
|  | *0212-Bacteroidetes(100);Bacteroidia(100);Bacteroidales(100);Porphyromonadaceae(100);Paludibacter(97);* | *B6*^+/-^ | 0.3074 | 0.0446 | 0.8453 |
|  | *0244-Bacteroidetes(100);Bacteroidia(100);Bacteroidales(100);Marinilabiaceae(76);Anaerophaga(76);* | *B6*^+/-^ | 0.3409 | 0.0087 | 0.5608 |
|  | *0316-Firmicutes(100);Clostridia(100);Clostridiales(100);Lachnospiraceae(100);Roseburia(77);* | *B6*^+/-^ | 0.2837 | 0.0095 | 0.5663 |
|  | *0325-Bacteroidetes(100);Bacteroidia(100);Bacteroidales(100);Prevotellaceae(100);Prevotella(71);* | *B6*^+/-^ | 0.3295 | 0.0327 | 0.8453 |
|  | *0384-Bacteroidetes(100);Bacteroidia(54);Bacteroidales(54);Marinilabiaceae(54);Anaerophaga(54);* | *B6*^+/-^ | 0.3145 | 0.0219 | 0.8453 |
|  | *0393-Bacteroidetes(100);Bacteroidia(54);Bacteroidales(54);* | *B6*^+/-^ | 0.3248 | 0.0462 | 0.8453 |
|  | *0394-Bacteroidetes(100);Bacteroidia(93);Bacteroidales(93);Rikenellaceae(93);Rikenella(93);* | *B6*^+/-^ | 0.4201 | 0.0092 | 0.5663 |
|  | *0433-Bacteroidetes(100);Bacteroidia(100);Bacteroidales(100);Marinilabiaceae(82);Anaerophaga(82);* | *B6*^+/-^ | 0.3466 | 0.0313 | 0.8453 |
|  | *0442-Bacteroidetes(100);Bacteroidia(100);Bacteroidales(100);Prevotellaceae(55);unclassified;* | *B6*^+/-^ | 0.3756 | 0.0147 | 0.7134 |
|  | *0443-Bacteroidetes(100);Bacteroidia(82);Bacteroidales(82);Porphyromonadaceae(82);Paludibacter(73);* | *B6*^+/-^ | 0.2247 | 0.0416 | 0.8453 |
|  | *0460-Bacteroidetes(100);Bacteroidia(100);Bacteroidales(100);Porphyromonadaceae(70);unclassified;* | *B6*^+/-^ | 0.2847 | 0.0471 | 0.8453 |
|  | *0463-Bacteroidetes(100);Bacteroidia(100);Bacteroidales(100);Bacteroidaceae(100);Bacteroides(100);* | *B6*^+/-^ | 0.3725 | 0.0198 | 0.8453 |
|  | *0496-Bacteroidetes(100);Bacteroidia(78);Bacteroidales(78);Rikenellaceae(78);Rikenella(78);* | *B6*^+/-^ | 0.4750 | 0.0007 | 0.2346 |
|  | *0502-Bacteroidetes(100);Bacteroidia(100);Bacteroidales(100);Porphyromonadaceae(100);Tannerella(100);* | *B6*^+/-^ | 0.3333 | 0.0496 | 0.8453 |
|  | *0528-Bacteroidetes(100);Bacteroidia(100);Bacteroidales(100);Porphyromonadaceae(88);Paludibacter(88);* | *B6*^+/-^ | 0.3922 | 0.0098 | 0.5663 |
|  | *0557-Bacteroidetes(100);Bacteroidia(100);Bacteroidales(100);Marinilabiaceae(88);Alkaliflexus(88);* | *B6*^+/-^ | 0.5000 | 0.0013 | 0.2607 |
|  | *0590-Bacteroidetes(100);Bacteroidia(86);Bacteroidales(86);Marinilabiaceae(58);Anaerophaga(58);* | *B6*^+/-^ | 0.3145 | 0.0466 | 0.8453 |
|  | *0630-Bacteroidetes(100);Sphingobacteria(100);Sphingobacteriales(100);Flammeovirgaceae(100);Limibacter(100);* | *B6*^+/-^ | 0.3540 | 0.0245 | 0.8453 |
|  | *0633-Bacteroidetes(100);Bacteroidia(100);Bacteroidales(100);Rikenellaceae(100);Alistipes(100);* | *B6*^+/-^ | 0.3333 | 0.0499 | 0.8453 |
|  | *0668-Bacteroidetes(100);Sphingobacteria(100);Sphingobacteriales(100);Flammeovirgaceae(100);Limibacter(100);* | *B6*^+/-^ | 0.3145 | 0.0466 | 0.8453 |
|  | *0696-Proteobacteria(100);BetaproteoBurkholderiales(100);Sutterellaceae(100);Parasutterella(100);* | *B6*^+/-^ | 0.3333 | 0.0453 | 0.8453 |
|  | *0769-Firmicutes(100);Clostridia(100);Clostridiales(100);Lachnospiraceae(100);Howardella(100);* | *B6*^+/-^ | 0.3131 | 0.0488 | 0.8453 |
|  | *0813-Bacteroidetes(100);Bacteroidia(100);Bacteroidales(100);Porphyromonadaceae(100);Paludibacter(100);* | *B6*^+/-^ | 0.3131 | 0.0472 | 0.8453 |
|  | *0856-Bacteroidetes(100);Bacteroidia(100);Bacteroidales(100);Rikenellaceae(61);Rikenella(61);* | *B6*^+/-^ | 0.3333 | 0.0499 | 0.8453 |
|  | *0906-Bacteroidetes(100);* | *B6*^+/-^ | 0.3333 | 0.0466 | 0.8453 |
|  | *0912-Firmicutes(100);Clostridia(100);Clostridiales(100);Ruminococcaceae(100);Oscillibacter(100);* | *B6*^+/-^ | 0.3333 | 0.0484 | 0.8453 |
|  | *0951-Bacteroidetes(100);FlavoFlavobacteriales(100);Flavobacteriaceae(100);Flagellimonas(75);* | *B6*^+/-^ | 0.3333 | 0.0430 | 0.8453 |
|  | *0992-Firmicutes(100);Clostridia(100);Clostridiales(100);Lachnospiraceae(100);Butyrivibrio(75);* | *B6*^+/-^ | 0.3333 | 0.0499 | 0.8453 |
|  | *1002-Firmicutes(100);Clostridia(100);Clostridiales(100);Ruminococcaceae(100);Pseudoflavonifractor(100);* | *B6*^+/-^ | 0.3333 | 0.0492 | 0.8453 |
|  | *1013-Bacteroidetes(100);Sphingobacteria(75);Sphingobacteriales(75);Flammeovirgaceae(75);Limibacter(75);* | *B6*^+/-^ | 0.3333 | 0.0498 | 0.8453 |
| 1 d.p.i. | *0002-Bacteroidetes(100);Bacteroidia(100);Bacteroidales(100);Rikenellaceae(95);Rikenella(95);* | *B6* ^-/-^ | 0.3264 | 0.0330 | 1.0000 |
|  | *0001-Proteobacteria(100);GammaproteoEnterobacteriales(100);Enterobacteriaceae(100);Salmonella(100);* | *B6*^+/-^ | 0.4243 | 0.0065 | 1.0000 |
|  | *0008-Proteobacteria(100);GammaproteoEnterobacteriales(100);Enterobacteriaceae(100);Salmonella(68);* | *B6*^+/-^ | 0.4241 | 0.0038 | 1.0000 |
|  | *0072-Firmicutes(100);Clostridia(100);Clostridiales(100);Clostridiaceae 1(100);Clostridium sensu stricto(100);* | *B6*^+/-^ | 0.3383 | 0.0282 | 1.0000 |
| before | *0012-Bacteroidetes(100);Sphingobacteria(95);Sphingobacteriales(95);Cytophagaceae(95);Meniscus(95);* | *RIII* - | 0.3034 | 0.0256 | 1.0000 |
| treatment | *0024-Bacteroidetes(100);Bacteroidia(96);Bacteroidales(96);Rikenellaceae(91);Rikenella(91);* | *RIII* - | 0.3546 | 0.0240 | 1.0000 |
|  | *0160-Bacteroidetes(100);Bacteroidia(100);Bacteroidales(100);Porphyromonadaceae(89);Tannerella(89);* | *RIII* - | 0.3725 | 0.0199 | 1.0000 |
|  | *0168-Bacteroidetes(100);Bacteroidia(66);Bacteroidales(66);Porphyromonadaceae(61);* | *RIII* - | 0.3351 | 0.0378 | 1.0000 |
|  | *0170-Bacteroidetes(100);Bacteroidia(95);Bacteroidales(95);Marinilabiaceae(88);Anaerophaga(88);* | *RIII* - | 0.3274 | 0.0284 | 1.0000 |
|  | *0324-Bacteroidetes(100);Bacteroidia(100);Bacteroidales(100);Prevotellaceae(100);Hallella(100);* | *RIII* - | 0.3588 | 0.0361 | 1.0000 |
|  | *0346-Bacteroidetes(100);Bacteroidia(87);Bacteroidales(87);Rikenellaceae(61);Rikenella(61);* | *RIII* - | 0.3171 | 0.0279 | 1.0000 |
|  | *0363-Bacteroidetes(100);Bacteroidia(100);Bacteroidales(100);Porphyromonadaceae(100);Paludibacter(93);* | *RIII* - | 0.3052 | 0.0315 | 1.0000 |
|  | *0010-Firmicutes(100);Erysipelotrichia(100);Erysipelotrichales(100);Erysipelotrichaceae(100);Turicibacter(100);* | *RIII*^+^ | 0.4010 | 0.0013 | 1.0000 |
|  | *0025-Firmicutes(100);Bacilli(100);Lactobacillales(100);Lactobacillaceae(100);Lactobacillus(100);* | *RIII*^+^ | 0.4979 | 0.0004 | 0.6703 |
|  | *0066-Firmicutes(100);Clostridia(100);Clostridiales(100);Lachnospiraceae(100);Robinsoniella(89);* | *RIII*^+^ | 0.3395 | 0.0406 | 1.0000 |
|  | *0118-Firmicutes(100);Clostridia(100);Clostridiales(100);Ruminococcaceae(52);* | *RIII*^+^ | 0.3155 | 0.0437 | 1.0000 |
|  | *0230-Bacteroidetes(100);Bacteroidia(100);Bacteroidales(100);Porphyromonadaceae(100);Paludibacter(86);* | *RIII*^+^ | 0.3399 | 0.0289 | 1.0000 |
|  | *0337-Firmicutes(100);Clostridia(100);Clostridiales(100);Lachnospiraceae(100);Robinsoniella(100);* | *RIII*^+^ | 0.2768 | 0.0149 | 1.0000 |
|  | *0378-Firmicutes(100);Erysipelotrichia(100);Erysipelotrichales(100);Erysipelotrichaceae(100);Allobaculum(70);* | *RIII*^+^ | 0.3780 | 0.0181 | 1.0000 |
|  | *0390-Bacteroidetes(100);Bacteroidia(100);Bacteroidales(100);Porphyromonadaceae(100);Paludibacter(93);* | *RIII*^+^ | 0.3940 | 0.0177 | 1.0000 |
|  | *0445-Firmicutes(100);Clostridia(100);Clostridiales(100);Lachnospiraceae(100);Robinsoniella(100);* | *RIII*^+^ | 0.3245 | 0.0175 | 1.0000 |
|  | *0447-Firmicutes(100);Clostridia(100);Clostridiales(100);Lachnospiraceae(100);Roseburia(100);* | *RIII*^+^ | 0.4001 | 0.0054 | 1.0000 |
|  | *0590-Bacteroidetes(100);Bacteroidia(86);Bacteroidales(86);Marinilabiaceae(58);Anaerophaga(58);* | *RIII*^+^ | 0.3560 | 0.0188 | 1.0000 |
|  | *0668-Bacteroidetes(100);SphingoSphingobacteriales(100);Flammeovirgaceae(100);Limibacter(100);* | *RIII*^+^ | 0.3560 | 0.0188 | 1.0000 |
|  | *1000-Bacteroidetes(100);Bacteroidia(100);Bacteroidales(100);Porphyromonadaceae(100);Barnesiella(75);* | *RIII*^+^ | 0.3216 | 0.0495 | 1.0000 |
| 1 d.p.i. | *0005-Firmicutes(100);Erysipelotrichia(100);Erysipelotrichales(100);Erysipelotrichaceae(100);Turicibacter(100);* | *RIII*^+^ | 0.2099 | 0.0142 | 1.0000 |
|  | *0136-Firmicutes(100);Clostridia(100);Clostridiales(100);Clostridiaceae 1(100);Clostridium sensu stricto(100);* | *RIII*^+^ | 0.2071 | 0.0067 | 1.0000 |
