# Supplementary material for: Expression of the Blood-Group-Related Gene B4galnt2 Alters Susceptibility to Salmonella Infection
Source: PLoS Pathog. 2015 Jul 2;11(7):e1005008. doi: 10.1371/journal.ppat.1005008 (PMC4489644; doi:10.1371/journal.ppat.1005008)
Supplement: S6 Table — (DOC) [file ppat.1005008.s017.doc]

| Indicator | Correlated genera | *ρ* | *P*-Value | *P*-Value (FDR) |
| --- | --- | --- | --- | --- |
| *Bacteroides* | *Parabacteroides* | -0.5811 | 0.0001 | 0.0025 |
|  | *Turicibacter* | -0.4847 | 0.0013 | 0.0198 |
|  | *Anaerotruncus* | 0.3992 | 0.0097 | 0.0728 |
|  | *Parasutterella* | 0.4042 | 0.0088 | 0.0728 |
|  | *Robinsoniella* | 0.4278 | 0.0053 | 0.0494 |
|  | *uncl. Alphaproteobacteria* | 0.4282 | 0.0052 | 0.0494 |
|  | *Odoribacter* | 0.4690 | 0.0020 | 0.0248 |
|  | *Prevotella* | 0.5452 | 0.0002 | 0.0043 |
|  | *uncl. Bacteroidales* | 0.5536 | 0.0002 | 0.0043 |
|  | *uncl. Prevotellaceae* | 0.9070 | <0.00001 | <0.00001 |
| *Parasutterella* | *Prevotella* | 0.4491 | 0.0032 | 0.0807 |
|  | *Anaerophaga* | 0.4809 | 0.0015 | 0.0547 |
|  | *uncl. Lactobacillales* | 0.4809 | 0.0015 | 0.0547 |
| *Prevotella* | *Parasutterella* | 0.4491 | 0.0032 | 0.0807 |
|  | *Bacteroides* | 0.5452 | 0.0002 | 0.0085 |
|  | *uncl. Prevotellaceae* | 0.6216 | <0.00001 | 0.0011 |
| *uncl. Prevotellaceae* | *Parabacteroides* | -0.6046 | <0.00001 | 0.0007 |
|  | *Turicibacter* | -0.4474 | 0.0034 | 0.0360 |
|  | *Anaerotruncus* | 0.4338 | 0.0046 | 0.0431 |
|  | *Odoribacter* | 0.4865 | 0.0013 | 0.0157 |
|  | *Robinsoniella* | 0.5249 | 0.0004 | 0.0064 |
|  | *uncl. Bacteroidales* | 0.5480 | 0.0002 | 0.0039 |
|  | *Prevotella* | 0.6216 | <0.00001 | 0.0005 |
|  | *Bacteroides* | 0.9070 | <0.00001 | <0.00001 |
| *Turicibacter* | *Bacteroides* | -0.4847 | 0.0013 | 0.0546 |
|  | *uncl. Prevotellaceae* | -0.4474 | 0.0034 | 0.0601 |
|  | *uncl. Erysipelotrichaceae* | 0.4398 | 0.0040 | 0.0601 |
|  | *Lactonifactor* | 0.4473 | 0.0034 | 0.0601 |
|  | *uncl. Porphyromonadaceae* | 0.4810 | 0.0015 | 0.0546 |
