# Supplementary material for: Expression of the Blood-Group-Related Gene B4galnt2 Alters Susceptibility to Salmonella Infection
Source: PLoS Pathog. 2015 Jul 2;11(7):e1005008. doi: 10.1371/journal.ppat.1005008 (PMC4489644; doi:10.1371/journal.ppat.1005008)
Supplement: S7 Table — (DOC) [file ppat.1005008.s018.doc]

| Time point | RDP9 Classification (modified by P. Schloss) | Abundance | *ρ* | *P*-Value | *P* (FDR) |
| --- | --- | --- | --- | --- | --- |
| before treatment | *uncl. Erysipelotrichaceae* | 5 | -0.3196 | 0.04169 | 0.5995 |
| 1 d.p.i. | *Acetanaerobacterium* | 9 | -0.3241 | 0.03869 | 0.1439 |
|  | *uncl. Bacteria* | 40 | -0.5697 | 0.00010 | 0.0010 |
|  | *uncl. Bacteroidales* | 5899 | -0.5598 | 0.00014 | 0.0010 |
|  | *uncl. Bacteroidetes* | 3032 | -0.6429 | 0.00001 | 0.0002 |
|  | *uncl. Clostridiales* | 92 | -0.3932 | 0.01098 | 0.0535 |
|  | *uncl. Firmicutes* | 59 | -0.5952 | 0.00004 | 0.0008 |
|  | *Parabacteroides* | 1349 | -0.4823 | 0.00141 | 0.0078 |
|  | *uncl. Porphyromonadaceae* | 691 | -0.5572 | 0.00015 | 0.0010 |
|  | *uncl. Ruminococcaceae* | 50 | -0.3401 | 0.02960 | 0.1283 |
|  | *Salmonella* | 17259 | 0.5685 | 0.00011 | 0.0010 |
|  | *Turicibacter* | 1165 | 0.3212 | 0.04059 | 0.1439 |
| before | *0195-Bacteroidetes(100);Bacteroidia(89);Bacteroidales(89);Porphyromonadaceae(89);Paludibacter(53);* | *34* | *-0.5176* | *0.00053* | *0.8241* |
| treatment | *0318-Firmicutes(100);Bacilli(100);Lactobacillales(100);Lactobacillaceae(100);Lactobacillus(100);* | *17* | *-0.4900* | *0.00115* | *0.8241* |
|  | *0620-Bacteroidetes(100);Bacteroidia(86);Bacteroidales(86);Rikenellaceae(86);Rikenella(86);* | *7* | *-0.4539* | *0.00287* | *0.8241* |
|  | *0312-Bacteroidetes(100);Bacteroidia(100);Bacteroidales(100);Porphyromonadaceae(95);Paludibacter(95);* | *17* | *-0.4499* | *0.00317* | *0.8241* |
|  | *0618-Bacteroidetes(100);Bacteroidia(86);Bacteroidales(86);* | *7* | *-0.3644* | *0.01915* | *0.8241* |
|  | *0406-Bacteroidetes(100);Bacteroidia(92);Bacteroidales(92);Porphyromonadaceae(59);Paludibacter(59);* | *12* | *-0.3640* | *0.01931* | *0.8241* |
|  | *0500-Bacteroidetes(100);Bacteroidia(100);Bacteroidales(100);Rikenellaceae(56);Rikenella(56);* | *9* | *-0.3568* | *0.02204* | *0.8241* |
|  | *0796-Bacteroidetes(100);Bacteroidia(100);Bacteroidales(100);Porphyromonadaceae(80);Paludibacter(61);* | *5* | *-0.3547* | *0.02287* | *0.8241* |
|  | *0273-Bacteroidetes(100);Bacteroidia(100);Bacteroidales(100);Marinilabiaceae(91);Anaerophaga(91);* | *22* | *-0.3510* | *0.02442* | *0.8241* |
|  | *0732-Bacteroidetes(100);Bacteroidia(100);Bacteroidales(100);Marinilabiaceae(84);Anaerophaga(84);* | *6* | *-0.3493* | *0.02517* | *0.8241* |
|  | *0692-Bacteroidetes(100);SphingoSphingobacteriales(100);Cytophagaceae(100);Meniscus(100);* | *6* | *-0.3416* | *0.02883* | *0.8241* |
|  | *0494-Bacteroidetes(100);Bacteroidia(100);Bacteroidales(100);Rikenellaceae(78);Rikenella(78);* | *9* | *-0.3386* | *0.03036* | *0.8241* |
|  | *0378-Firmicutes(100);Erysipelotrichia(100);Erysipelotrichales(100);Erysipelotrichaceae(100);Allobaculum(70);* | *13* | *-0.3383* | *0.03050* | *0.8241* |
|  | *0320-Bacteroidetes(100);Bacteroidia(100);Bacteroidales(100);Porphyromonadaceae(77);* | *17* | *-0.3257* | *0.03770* | *0.8241* |
|  | *0730-Bacteroidetes(100);Bacteroidia(100);Bacteroidales(100);Porphyromonadaceae(100);Paludibacter(100);* | *6* | *-0.3233* | *0.03924* | *0.8241* |
|  | *0634-Firmicutes(100);Clostridia(100);Clostridiales(100);Lachnospiraceae(100);Robinsoniella(100);* | *7* | *-0.3120* | *0.04706* | *0.8241* |
|  | *0217-Bacteroidetes(100);Bacteroidia(94);Bacteroidales(94);Porphyromonadaceae(73);Tannerella(73);* | *29* | *-0.3103* | *0.04833* | *0.8241* |
|  | *0809-Firmicutes(100);Clostridia(100);Clostridiales(100);Lachnospiraceae(100);Robinsoniella(100);* | *5* | *0.3089* | *0.04941* | *0.8241* |
|  | *0840-Firmicutes(100);Clostridia(100);Clostridiales(100);Ruminococcaceae(80);Butyricicoccus(80);* | *5* | *0.3089* | *0.04941* | *0.8241* |
|  | *0099-Firmicutes(100);Clostridia(100);Clostridiales(100);Ruminococcaceae(100);Oscillibacter(99);* | *94* | *0.3168* | *0.04360* | *0.8241* |
|  | *0586-Firmicutes(100);Clostridia(100);Clostridiales(100);Lachnospiraceae(100);Clostridium XlVa(86);* | *7* | *0.3181* | *0.04271* | *0.8241* |
|  | *0272-Firmicutes(100);Clostridia(100);Clostridiales(100);Lachnospiraceae(100);Robinsoniella(96);* | *22* | *0.3210* | *0.04070* | *0.8241* |
|  | *0451-Firmicutes(100);Clostridia(100);Clostridiales(100);Ruminococcaceae(100);Anaerotruncus(91);* | *11* | *0.3213* | *0.04051* | *0.8241* |
|  | *0460-Bacteroidetes(100);Bacteroidia(100);Bacteroidales(100);Porphyromonadaceae(70);* | *10* | *0.3253* | *0.03797* | *0.8241* |
|  | *0432-Firmicutes(100);Clostridia(100);Clostridiales(100);Lachnospiraceae(100);Robinsoniella(82);* | *11* | *0.3267* | *0.03710* | *0.8241* |
|  | *0640-Firmicutes(100);Clostridia(100);Clostridiales(100);Lachnospiraceae(100);Lachnospiracea incertae sedis(86);* | *7* | *0.3470* | *0.02625* | *0.8241* |
|  | *0693-Firmicutes(100);Clostridia(100);Clostridiales(100);Lachnospiraceae(100);Catonella(100);* | *6* | *0.3483* | *0.02563* | *0.8241* |
|  | *0313-Firmicutes(100);Clostridia(100);Clostridiales(100);Lachnospiraceae(100);Anaerostipes(83);* | *17* | *0.3543* | *0.02302* | *0.8241* |
|  | *0343-Firmicutes(100);Clostridia(100);Clostridiales(100);Lachnospiraceae(100);Robinsoniella(87);* | *15* | *0.3555* | *0.02255* | *0.8241* |
|  | *0772-Firmicutes(100);Clostridia(100);Clostridiales(100);Ruminococcaceae(100);Pseudoflavonifractor(100);* | *5* | *0.3634* | *0.01953* | *0.8241* |
|  | *0065-Firmicutes(100);Clostridia(100);Clostridiales(100);Lachnospiraceae(100);Robinsoniella(87);* | *200* | *0.3744* | *0.01587* | *0.8241* |
|  | *0226-Firmicutes(100);Clostridia(100);Clostridiales(100);Lachnospiraceae(100);Blautia(97);* | *27* | *0.3747* | *0.01580* | *0.8241* |
|  | *0042-Firmicutes(100);Clostridia(100);Clostridiales(100);Lachnospiraceae(100);Robinsoniella(98);* | *398* | *0.4456* | *0.00351* | *0.8241* |
| 1 d.p.i. | *0087-Bacteroidetes(100);Bacteroidia(100);Bacteroidales(100);Rikenellaceae(62);Rikenella(62);* | *127* | *-0.5791* | *0.00007* | *0.0113* |
|  | *0111-Bacteroidetes(100);Bacteroidia(100);Bacteroidales(100);Rikenellaceae(75);Rikenella(75);* | *82* | *-0.5640* | *0.00012* | *0.0128* |
|  | *0002-Bacteroidetes(100);Bacteroidia(100);Bacteroidales(100);Rikenellaceae(95);Rikenella(95);* | *13695* | *-0.5510* | *0.00019* | *0.0147* |
|  | *0122-Bacteroidetes(100);Bacteroidia(100);Bacteroidales(100);Porphyromonadaceae(100);Barnesiella(99);* | *65* | *-0.4986* | *0.00091* | *0.0565* |
|  | *0142-Bacteroidetes(100);Bacteroidia(100);Bacteroidales(100);Rikenellaceae(83);Rikenella(83);* | *51* | *-0.4550* | *0.00280* | *0.1245* |
|  | *0032-Firmicutes(100);Bacilli(98);Lactobacillales(98);Carnobacteriaceae(98);Isobaculum(86);* | *604* | *-0.4462* | *0.00346* | *0.1245* |
|  | *0278-Bacteroidetes(100);Bacteroidia(72);Bacteroidales(72);Porphyromonadaceae(62);Paludibacter(58);* | *21* | *-0.4458* | *0.00349* | *0.1245* |
|  | *0013-Bacteroidetes(100);Bacteroidia(94);Bacteroidales(94);Porphyromonadaceae(87);Barnesiella(66);* | *2084* | *-0.4444* | *0.00360* | *0.1245* |
|  | *0068-Bacteroidetes(100);Bacteroidia(100);Bacteroidales(100);Porphyromonadaceae(100);Barnesiella(98);* | *181* | *-0.4096* | *0.00782* | *0.2254* |
|  | *0176-Bacteroidetes(80);* | *39* | *-0.4045* | *0.00872* | *0.2254* |
|  | *0077-Bacteroidetes(100);Bacteroidia(75);Bacteroidales(75);Porphyromonadaceae(51);* | *163* | *-0.4013* | *0.00931* | *0.2254* |
|  | *0006-Bacteroidetes(100);Bacteroidia(95);Bacteroidales(95);Porphyromonadaceae(91);Paludibacter(87);* | *4936* | *-0.4007* | *0.00942* | *0.2254* |
|  | *0015-Bacteroidetes(100);Bacteroidia(100);Bacteroidales(100);Porphyromonadaceae(98);Paludibacter(98);* | *1762* | *-0.3683* | *0.01782* | *0.3198* |
|  | *0017-Bacteroidetes(100);Bacteroidia(100);Bacteroidales(100);Porphyromonadaceae(100);Parabacteroides(100);* | *1590* | *-0.3675* | *0.01811* | *0.3198* |
|  | *0240-Bacteroidetes(100);Bacteroidia(100);Bacteroidales(100);Porphyromonadaceae(96);Parabacteroides(80);* | *25* | *-0.3596* | *0.02093* | *0.3198* |
|  | *0083-Firmicutes(100);Bacilli(95);Lactobacillales(95);Carnobacteriaceae(95);Isobaculum(95);* | *136* | *-0.3576* | *0.02173* | *0.3198* |
|  | *0132-Firmicutes(100);Bacilli(75);Lactobacillales(75);Carnobacteriaceae(75);Isobaculum(75);* | *58* | *-0.3576* | *0.02173* | *0.3198* |
|  | *0354-Bacteroidetes(100);Bacteroidia(100);Bacteroidales(100);Porphyromonadaceae(100);Paludibacter(100);* | *15* | *-0.3546* | *0.02292* | *0.3198* |
|  | *0108-Bacteroidetes(100);Bacteroidia(100);Bacteroidales(100);Porphyromonadaceae(93);Tannerella(75);* | *88* | *-0.3545* | *0.02295* | *0.3198* |
|  | *0026-Firmicutes(100);Clostridia(100);Clostridiales(100);Clostridiaceae 4(61);Geosporobacter(61);* | *724* | *-0.3528* | *0.02365* | *0.3198* |
|  | *0119-Bacteroidetes(100);Bacteroidia(100);Bacteroidales(100);Porphyromonadaceae(73);Paludibacter(73);* | *68* | *-0.3482* | *0.02569* | *0.3328* |
|  | *0071-Firmicutes(100);Bacilli(99);Lactobacillales(99);Carnobacteriaceae(99);Pisciglobus(97);* | *173* | *-0.3413* | *0.02897* | *0.3604* |
|  | *0430-Bacteroidetes(100);Bacteroidia(100);Bacteroidales(100);Porphyromonadaceae(82);Paludibacter(82);* | *11* | *-0.3311* | *0.03446* | *0.3759* |
|  | *0274-Bacteroidetes(100);Bacteroidia(100);Bacteroidales(100);Porphyromonadaceae(100);Parabacteroides(77);* | *21* | *-0.3305* | *0.03483* | *0.3759* |
|  | *0023-Bacteroidetes(100);Bacteroidia(99);Bacteroidales(99);Porphyromonadaceae(99);Paludibacter(96);* | *839* | *-0.3304* | *0.03485* | *0.3759* |
|  | *0450-Bacteroidetes(100);Bacteroidia(55);Bacteroidales(55);Rikenellaceae(55);Rikenella(55);* | *11* | *-0.3265* | *0.03724* | *0.3782* |
|  | *0322-Bacteroidetes(100);Bacteroidia(77);Bacteroidales(77);Rikenellaceae(59);Rikenella(59);* | *17* | *-0.3107* | *0.04804* | *0.3782* |
|  | *0005-Firmicutes(100);Erysipelotrichia(100);Erysipelotrichales(100);Erysipelotrichaceae(100);Turicibacter(100);* | *5193* | *0.3724* | *0.01649* | *0.3198* |
|  | *0001-ProteoGammaproteoEnterobacteriales(100);Enterobacteriaceae(100);Salmonella(100);* | *15356* | *0.5894* | *0.00005* | *0.0113* |
